# Regional differences in water beetle communities networks settling in dystrophic lakes in northern Poland

**DOI:** 10.1038/s41598-023-39689-z

**Published:** 2023-08-05

**Authors:** Joanna Pakulnicka, Marek Kruk

**Affiliations:** 1 Department of Ecology and Environmental Protection, University of Warmia and Mazury in Olsztyn, Lodzki sq. 3, 10–727 Olsztyn, Poland; 2 Department of Mathematical Modelling and Applied Informatics, University of Warmia and Mazury in Olsztyn, Sloneczna 54, 10–719 Olsztyn, Poland

**Keywords:** Statistical methods, Biodiversity, Community ecology, Conservation biology, Ecological networks, Evolutionary ecology, Freshwater ecology, Macroecology, Computational biology and bioinformatics, Ecology

## Abstract

The relationships between the species that form the networks in small dystrophic lakes remain poorly recognised. To investigate and better understand the functioning of beetle communities in different ecosystems, we created three network models that we subjected to graph network analysis. This approach displays correlation–based networks of connections (edges) between objects (nodes) by evaluating the features of the whole network and the attributes of nodes and edges in the context of their roles, expressed by centrality metrics. We used this method to determine the importance of specific species in the networks and the interspecific relationships. Our analyses are based on faunal material collected from 25 dystrophic lakes in three regions of northern Poland. We found a total of 104 species representing different ecological elements and functional trophic groups. We have shown that the network of relationships between the biomass of species differs considerably in the three study regions. The Kashubian Lakeland had the highest cohesion and density, while the network in the Suwalki Lakeland was the thinnest and most heterogeneous, which might be related to the fractal structure and the degree of development of the studied lakes. Small–bodied predators that congregated in different clusters with species with similar ecological preferences dominated all networks. We found the highest correlations in the Masurian Lakeland, where we obtained the highest centralisation of the network. Small tyrphophiles typically occupied the central places in the network, while the periphery of the network consisted of clusters with different habitat preferences, including large predators. The species that were most important for network cohesion and density were mainly tyrphophilous species, such as *Anacaena lutescens*, *Hygrotus decoratus*, *Enochrus melanocephalus* and *Hydroporus neglectus*. The values of attributes determining the role of species in community networks were influenced by both biotic and environmental factors.

## Introduction

The extensive literature on hydrobiology is dominated by publications in which much attention is paid to the structure of biocenoses and the relationships between organism communities and specific environmental conditions^1–11^. In contrast, organism communities are relatively rarely studied from a functional perspective, as an intricate network of interactions in a broadly defined predator–prey system in which biomass flows^12,13^. The structure and functioning of networks between different species in freshwater has hardly been studied yet^13^. It is clear that invertebrates, including beetles, are the dominant component of trophic networks of aquatic ecosystems^13,14^. They are a group of organisms that are highly diverse in terms of species and ecology, are widespread in the environment and typically occur in large numbers in very different habitats^15^. The literature shows that beetles use a pool of nutrients from different trophic levels, although most beetles are predatory, which underlines their important role in trophic networks as regulators of the numbers of other organisms^13,14,16,17^. The classification of beetles into specific functional groups, indicating not only the type of food consumed but also the way food is obtained, is extremely important for a more comprehensive analysis of trophic networks^12,18,19^.

The application of graph theory–based network analysis is becoming increasingly popular as a research tool in aquatic ecosystem ecology, as it provides greater insight into biocoenosis structure, formation, trophic functioning and responses to changing environmental conditions, especially when modelling is based on data collected in the field^12,13,20–23^. Such studies also offer a different approach to a species that is not just another element of a network, but also a representative of interactive multidimensionality throughout the ecosystem^24^.

We based the relationships between species in the network on the correlations between biomasses of beetle taxa. This approach is most relevant for trophic functional analysis of organismal communities^25^. It allows the construction of the trophic structure of biocenoses and the assessment of the influence of rare species on the stability of the network^22^. The network analysis allows to conduct detailed studies even on less charismatic and non–model species, which often have a small body size, which can help identify several unexpected but potentially important trophic links in the network^13,16,17,26,27^. Some authors^13^ point out that species network structures are less known in smaller water bodies than in lakes or rivers, so further studies are warranted^12^. This network–oriented approach allows for a more comprehensive recognition and understanding of how groups of organisms function in different ecosystems and helps to more accurately predict the adaptation of biocenotic systems to changing environmental conditions^21,28^. This is particularly important in the context of ongoing climate change, threats to habitats from humans and the search for ways to restore already.

Dystrophic (humic) lakes, which constitute for 5% of all lakes in Poland^29–31^, are a particularly endangered and vulnerable element of ecological landscapes in Europe. This type of lake is characteristic of the boreal zone and occurs only sporadically in the lowlands of Central Europe^32^. In Poland, dystrophic lakes are most numerous in the northern part of the country (lowlands), inside the forest areas. They fill cirques formed by the melting of dead ice blocks left by the retreating glacier during the last interglacial. Although the formation of all dystrophic lakes is similar, there are significant differences between regions related to the time period when the glacier retreated. According to Marks^33^, the boundary of the Late Vistulian maximum ice sheet in Poland was temporally overlapping and generally younger towards the east, which would mean that the lakes in the western part of northern Poland are ecologically younger. Dystrophic (humic) lakes are characterised by low pH, very low calcium content in water and sediment, peat–covered catchments, spreading floating *Sphagnum* mats on the water surface, low phytoplankton biomass, low species diversity and respiration rates higher than primary production^32,34,35^. Despite their common origin, dystrophic lakes represent different successional stages in which their surface area gradually decreases and the lakes become shallower due to the lowered water level and accumulation of biogenic sediments. The characteristics of a floating peat mat in the littoral zone (width, length and compactness) can indicate the ecological age of the lake^36^. The natural ageing of lakes progresses very slowly if the catchment areas of the lakes are natural or exposed to only minor anthropogenic influences^37^.

The accessibility of nutrients (production) in lakes in the natural environment depends on temperature and dystrophic lakes can respond to fluctuations in climatic conditions as well as local and global anthropic pressures^32,37^. Among the anthropogenic factors that significantly accelerate the natural succession of dystrophic lakes, the use of the catchment area should be indicated, i.e. its drainage, deforestation and peat mining, which lead to a deterioration of water quality^6,31,32,37,38^.

The littoral is always the part of a lake that is most sensitive to the influence of external factors^5,38^. It is also the area where macroorganisms, including beetles, occur in high species diversity. Many of them are specialised species that react more sensitively and quickly to changes in the environment. Knowledge of the ecological preferences of beetles can be used in monitoring aquatic ecosystems in line with the Water Framework Directive^7,39^.

The main objectives of our study were to (1) compare the characteristics of the network of interspecific relationships in beetle communities in lakes in different regions of the country, (2) determine the importance of specific species and mutualistic relationships between them in these networks, and (3) identify the role of different ecological and functional elements in the character of the network in specific regions.

To achieve these goals, we formulated the following working hypotheses: H1: assemblages of beetles differ in the selected regions with respect to network attributes; H2: regardless of these differences, beetles are dominated by mutually positive relationships indicating co–occurrence, lack of major competition and a food base derived from other groups of aquatic organisms; H3: species differ with particular importance for network stability in the regions; H4: networks tend to divide into clusters consisting of species with similar habitat and food preferences; H5: biotic and environmental factors influence species attribute values in community networks.

## Results

### General characteristics of the collected material

The collected material contains a total of 4533 beetles representing 104 species (Table 1, Table S1). The species diversity in the samples collected from the lakes studied ranged from 1 to 25. The most species (73) we found in the Masurian Lakeland, where the highest number of beetles was also collected. The least numerous and species–rich material (54 species) came from the Kashubian Lakes (Pomeranian Lakeland).

**Table 1:** General characteristics of the material.

Parameter	Kashubian Lakeland (n = 71)		Mazurian Lakeland (n = 66)			Suwalki Lakeland (n = 70)		
	N	$\overline{\mathrm{x} }$ ± SD	N	$\overline{\mathrm{x} }$ ± SD		N	$\overline{\mathrm{x} }$ ± SD	
Abundance	695	2.76 ± 5.53	1937	4.18 ± 9.51		1901	3.66 ± 6.03	
Numer of species	54 (1–12)		73 (1–28)			68 (1–17)		
Index H′	2.75		2.67			2.69		
Biomass	19,131.44	76.1 ± 258.02	84,691.67	182.9 ± 926.56		37,957.71	73.14 ± 259.04	
Dominant species	*Noterus crassocornis*		*Noterus crassocornis*			*Noterus crassocornis*		
	*Anacaena lutescens*		*Anacaena lutescens*			*Anacaena lutescens*		
	*Helochares griseus*		*Hydroporus tristis*			*Hydroporus tristis*		
	*Hydroporus palustris*		*Enochrus affinis*			*Enochrus affinis*		
Species with the highest individual biomass	*Dytiscus dimidiatus*		*Cybister lateralimarginalis*			*Dytiscus marginalis*		
	*Dytiscus marginalis*		*Dytiscus marginalis*			*Hydrochara caraboides*		
	*Dytiscus lapponicus*		*Dytiscus lapponicus*			*Acilius sulcatus*		
	*Acilius canaliculatus*		*Colymbetes paykulli*			*Acilius canaliculatus*		
	*Ilybius fenestratus*		*Acilius sulcatus*			*Graphoderus bilineatus*		
	*Hydaticus seminiger*		*Acilius canaliculatus*			*Graphoderus cinereus*		
Ecological groups – N, (S)								
Eurybionts	396 (29)	2.93 ± 5.56	1084 (35)		4.53 ± 11.22	986 (39)		4.12 ± 6.75
Tyrphophiles	252 (17)	2.89 ± 6.28	807 (27)		4.20 ± 7.76	796 (20)		3.42 ± 6.75
Argilophiles	8 (4)	1.33 ± 0.51	22 (6)		1.22 ± 0.54	110 (7)		2.82 ± 5.1
Lake and river species	39 (4)	1.69 ± 1.49	24 (5)		1.71 ± 1.27	9 (2)		1.12 ± 0.36
Functional groups – N, (S)								
Shredders	314 (16)	2.93 ± 5.93	717 (21)		3.56 ± 6.46	784 (20)		3.37 ± 5.51
Grazers and scraper	1 (1)	1.00	18 (3)		1.64 ± 1.29	17 (2)		1.88 ± 1.76
Predators	379 (36)	4.87 ± 1.29	1193 (46)		4.87 ± 11.65	1097 (43)		3.98 ± 6.53
Polyphaga	1 (1)	1.00	9 (3)		1.5 ± 0.84	3 (3)		1.0 ± 0.00

N abundance, S number of species, $\overline{\mathrm{x} }$ mean, *SD* standard deviation, H’ Shannon–Wiener index, n number of samples.

The species richness determined in the lakes studied differed statistically significantly in the individual regions (Kruskal–Wallis test: H(2, N = 207) = 54.03, p = 0.00001 (Table 2). Significant differences were also found in the number (Kruskal–Wallis test: H(2, N = 207) = 56.41, p = 0.00001) and biomass of beetles (Kruskal–Wallis test: H(2, N = 207) = 32.40, p = 0.00001). Significant statistical differences between the regions studied (p – value for multiple comparisons) are listed in Table 2.

**Table 2:** Differences between parameters in highlighted regions (KL Kashubian Lakeland, ML Mazurian Lakeland, SW Suwalki Lakeland).

Parameter	H	df	p	Post–hoc (p)
Abundance (N)	56.41	2, N = 207	0.00001	KL – ML (0.00000032) KL – SL (0.0000001)
Number of species (S)	23.02	2, N = 207	0.0000001	KL – ML (0.00000006) KL – SL (0.00000022)
Index H′ (Shannon–Wiener index)	10.15	2, N = 207	0.53	
Biomass	4.69	2, N = 207	0.0001	KL – ML (0.0000003) ML – SL (0.000003)
Ecological groups (N, S)				
Eurybionts	33.44	2, N = 207	0.00001	KL – ML (0.00000032) KL – SL (0.00000001)
	29.32	2, N = 207	0.000001	KL – ML (0.0002) KL – SL (0.00000082)
Tyrphophiles	45.81	2, N = 207	0.00001	KL – ML (0.0000001) KL – SL (0.00000006)
	50.44	2, N = 207	0.00001	KL – ML (0.0000006) KL – SL (0.000000008)
Argilophiles	3.96	2, N = 207	0.026	KL – ML (0.02) ML – SL (0.04)
	22.43	2, N = 207	0,00,000,002	KL – ML (0.29) ML – SL (0.04)
Lake and river species	7.78	2, N = 207	0.02	KL – ML (0.02)
	7.74	2, N = 207	0.03	KL – SL (0.04)
Functional groups (N, S)				
Shredders	30.72	2, N = 207	0.000001	KL – ML (0.0000001) KL – SL (0.00002)
	33.69	2, N = 207	0.000001	KL – ML (0.0000001) KL – SL (0.00002)
Grazers and scrapers	7.87	2, N = 207	0.02	KL – ML (0.0000001) KL – SL (0.00002)
	7.85	2, N = 207	0.02	KL – ML (0.0000001) KL – SL (0.00002)
Predators	51.70	2, N = 207	0.00001	KL – ML (0.00000002) KL – SL (0.0000000003)
	40.19	2, N = 207	0.00001	KL – ML (0.0000001) KL – SL (0.00002)
Polyphaga	3.18	2, N = 207	0.20	
	3.15	2, N = 207	0.21	

Results of ANOVA Kruskal – Wallis test: H (statistics), df degree of freedom, p p value, N abundance, S number of species.

**Figure 1:**
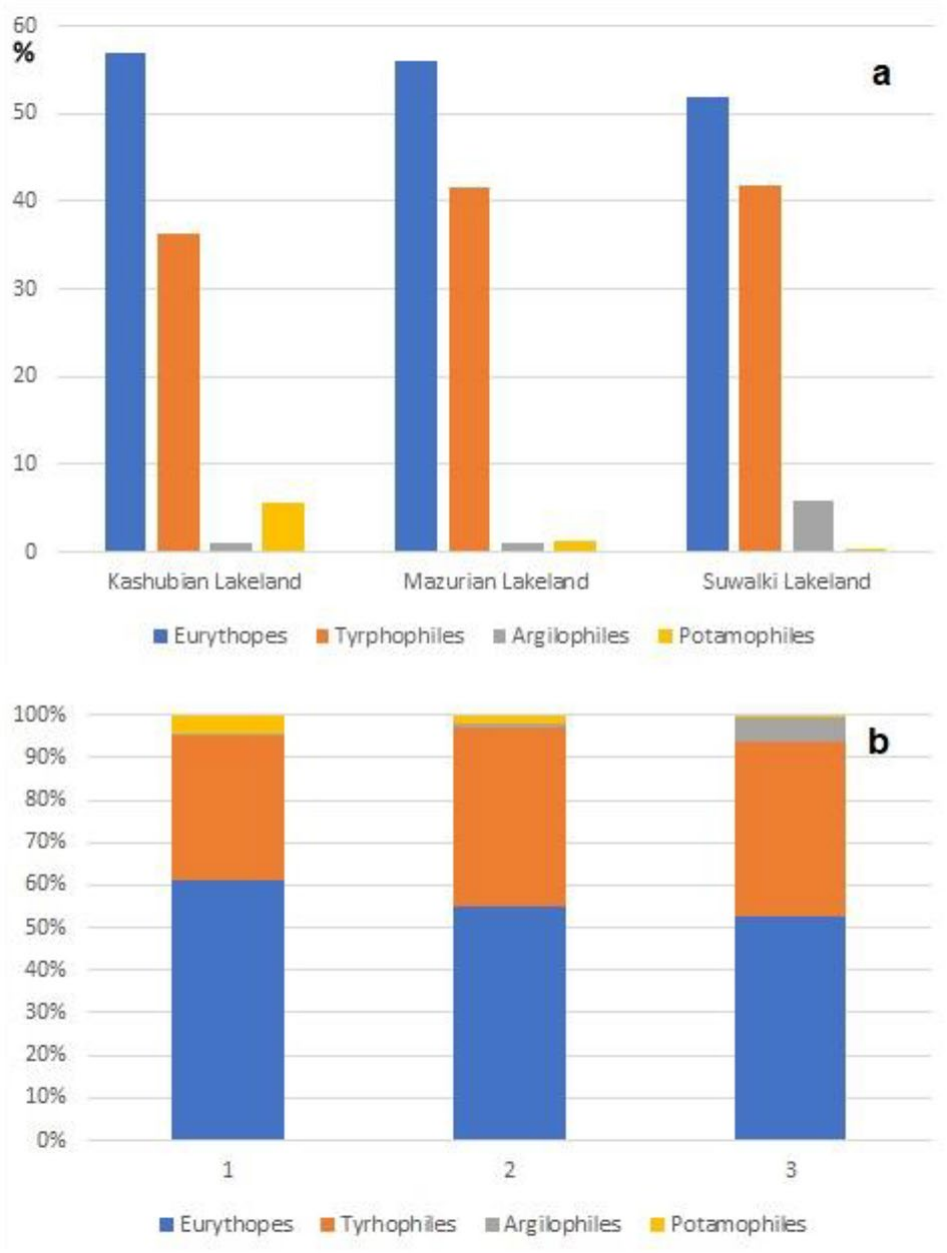
The ecological structure of water beetles (**a**) in lakes of the three regions and (**b**) in different stage of succession (1 – oligohumic, 2 – mesohumic, 2 – polyhumic lakes). Percentage share of distinguished synecological groups in lakes.

The most numerous species in the collected material were *Noterus crassicornis* (20.36%), *Anacaena lutescens* (17.1%), followed by *Enochrus affinis* (4.41%), *Helochares grisus* (4.23%) and *Enochrus coarctatus* (4.12%). The species with the highest individual biomass were *Dytiscus dimidiatus*, *D. marginalis*, *D. lappoonicus*, *Acilius canaliculatus*, *A. sulcatus* and *Graphoderus cinereus* (Table 2). The core of the collected fauna was formed by eurybionts (54.4%) and tyrphophiles (0.40%), which were most numerous in the lakes (Fig. 1).

These assemblages were also characterised by the highest species diversity. The remaining insect fauna consisted of argilophiles (3.1%) and lake and river species (potamophiles) (1.6%). The ecological structures of the fauna differ in the individual lakes. The most specific element of the fauna, tyrphophiles, were most numerous in the Suwalki Lakeland (41.9% of all beetles) and in the Masurian Lakeland (41.7%), while their share in the total number of beetles in the lakes of the Kashubian Lakeland was 36.2%. Argilophiles were most numerous in polyhumic lakes, especially in the Suwalki Lakeland (5.8%), while their percentage share was about the same in the other two regions (1.1%). The potamophilous species, in turn, were most abundant in the oligohumic lakes, especially in the Kashubian lakes (5.6%) and least numerous in the Suwalki Lakeland (0.4%). All distinguished species groups showed significant regional differences in species richness and abundance (Fig. 1) (Table 2).

**Figure 2:**
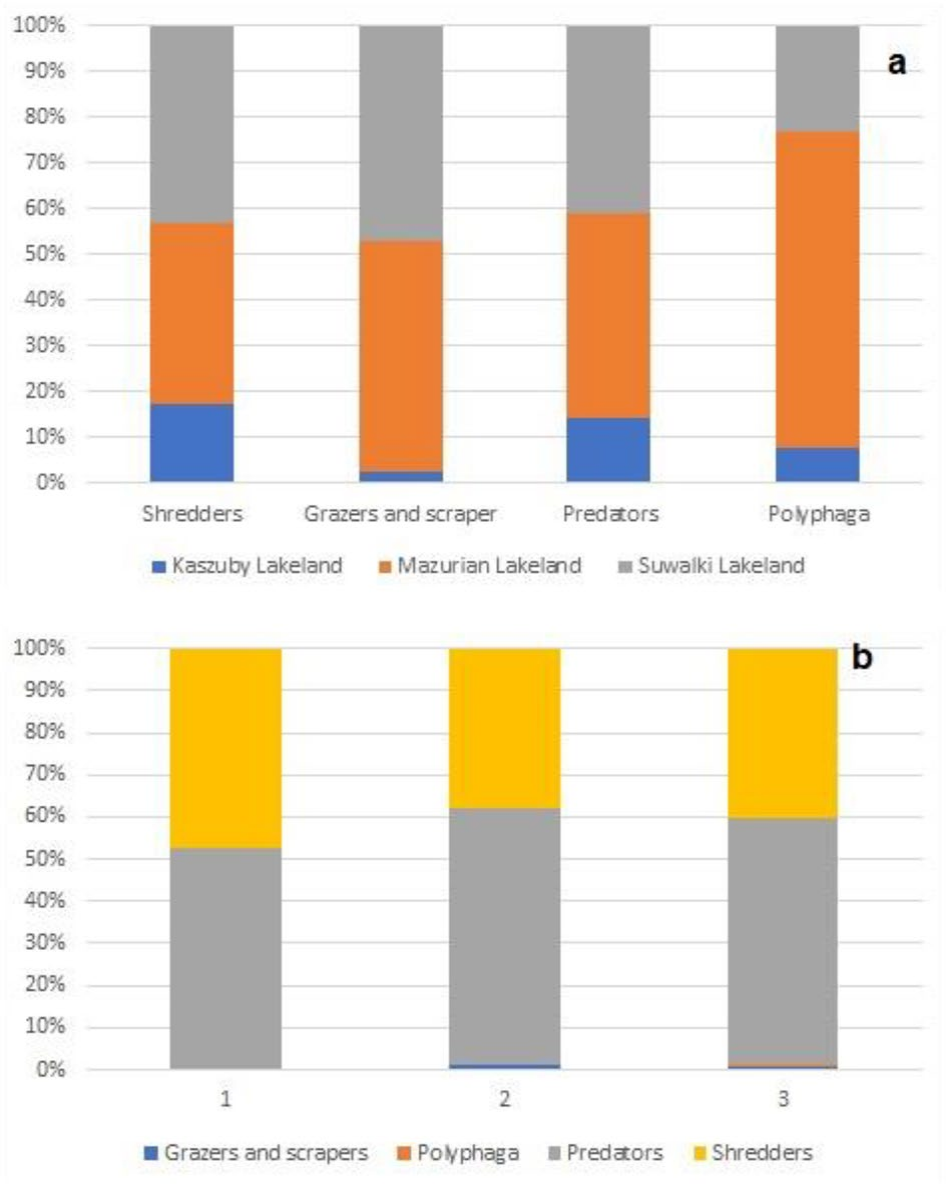
Functional feeding groups of aquatic beetles in lakes (**a**) in lakes of the three regions and (**b**) in different stage of succession (1 – oligohumic, 2 – mesohumic, 2 – polyhumic lakes). Percentages distinguished functional groups in lakes.

Predators (57.7%) and shredders (41.2%) dominated quantitatively the trophic structure of the collected material. The other functional groups, i.e. grazers and scrapers (0.89%) and polyphages (0.15%), had a negligible share in the beetle fauna (Fig. 2). We found far more polyphaga in the Masurian Lakeland (usually in mesohumic lakes) than in the other two lake areas. Table 2 listed significant statistical differences between the regions studied (p–value for multiple comparisons).

### Network structure

The network of interactions between the beetles in the regions compared differed considerably in terms of the key attributes used to describe them. These were attributes describing the networks: the number of neighbours, the nearest path, the clustering coefficient, the network centralisation, the network density and the network heterogeneity. We used the following metrics to characterise the role of specific nodes (species) and connections between them: node degree centrality (NDC), node closeness centrality (NCC), node betweenness centrality (NBC), clustering coefficient, correlation coefficient between neighbouring nodes (R) and edge betweenness centrality (EBC).

**Figure 3:**
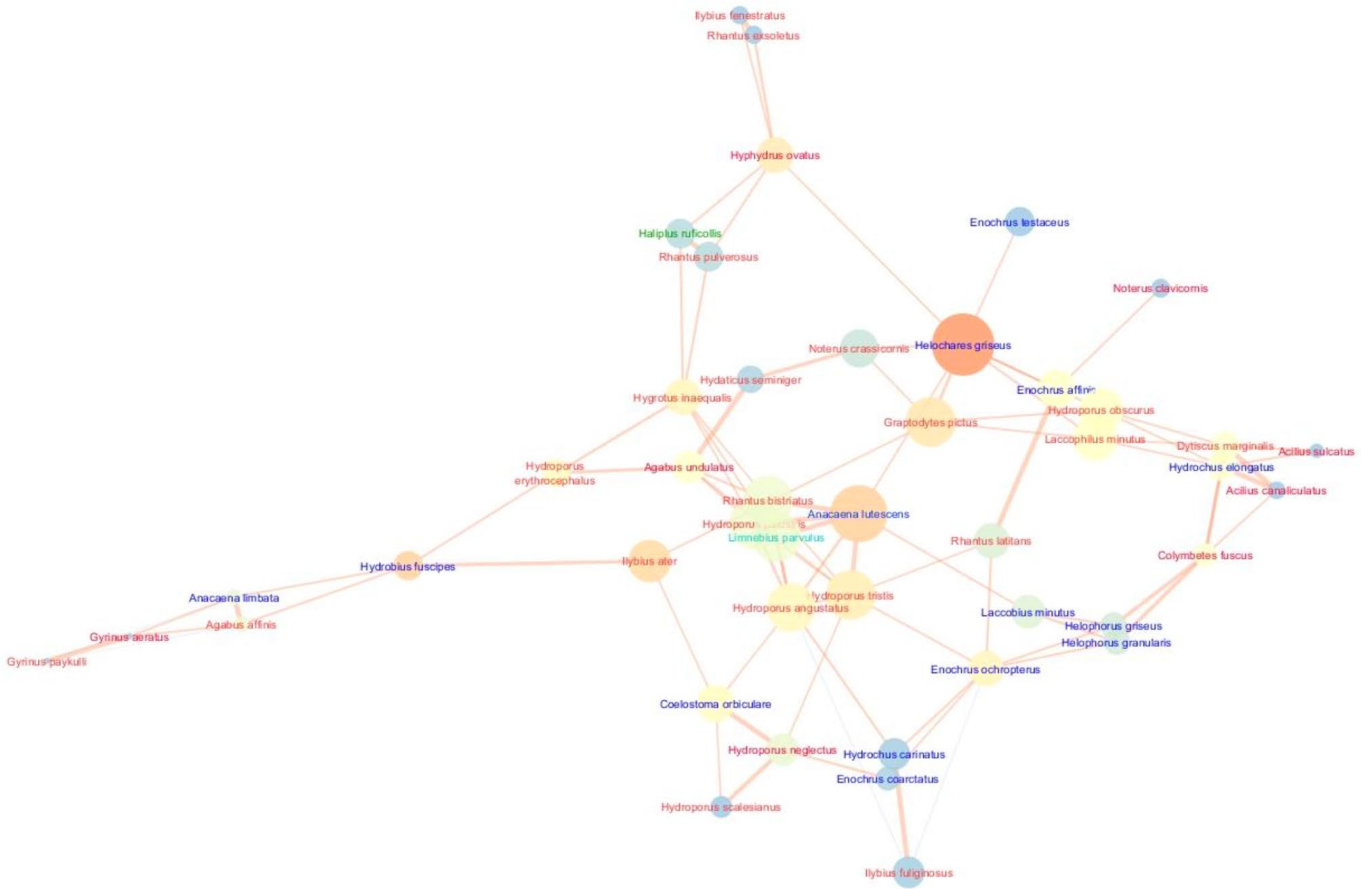
Network graph of the interactions between beetles species in the Kashubian Lakeland with node closeness centrality (NCC), node betweenness centrality (NBC) and correlation coefficient (R). Node size is proportional to the NCC measure; node colour ranging from blue (dark) to orange (bright) is proportional to the NBC measure; edge thickness is proportional to the R correlation coefficient. Sign of the relationship: a bright orange edge denotes positive relations between nodes, while a dark blue edge represents negative relations. The font color means: red - predators, blue - shredders, dark green - polyphages, light green - grazers and scrapers. We generated the graph using the Metscape application in Cytoscape 3.7.2 package, https://cytoscape.org.

The analysed network in the Pomeranian (Kashubian) Lakeland was characterised by the highest cohesion and density, expressed by clustering (0.461) and density (0.095) coefficients (Fig. 3; Table 3).

**Table 3:** General attributes of the water beetles network in compared regions.

Atribute	Region		
	Kashubian Lakeland	Mazurian Lakeland	Suwalki Lakeland
Clustering coefficient	0.461	0.277	0.234
Network centralization	0.091	0.107	0.077
Shortest paths (100%)	1980	4830	4422
Characteristic path length	3.463	3.332	3.670
Average numer of neighbours	4.178	4.829	4.09
Network density	0.095	0.070	0.062
Network heterogeneity	0.458	0.572	0.453

This means the highest number of direct and indirect links between species. At the same time, we observed a moderate value of the centrality metric (0.091), as well as the average number of neighbours (4.178) per species (node), i.e. the number of interspecific interactions, and the values of the parameters describing the communication pathways between taxa. In the Pomeranian region, we found the shortest paths (1980), indicating the lowest number of highest correlations between species in the network, as well as a relatively low value of the so–called characteristic path length (4.178), indicating the presence of taxa communicating with the highest number of species (Fig. 3; Table 3).

**Figure 4:**
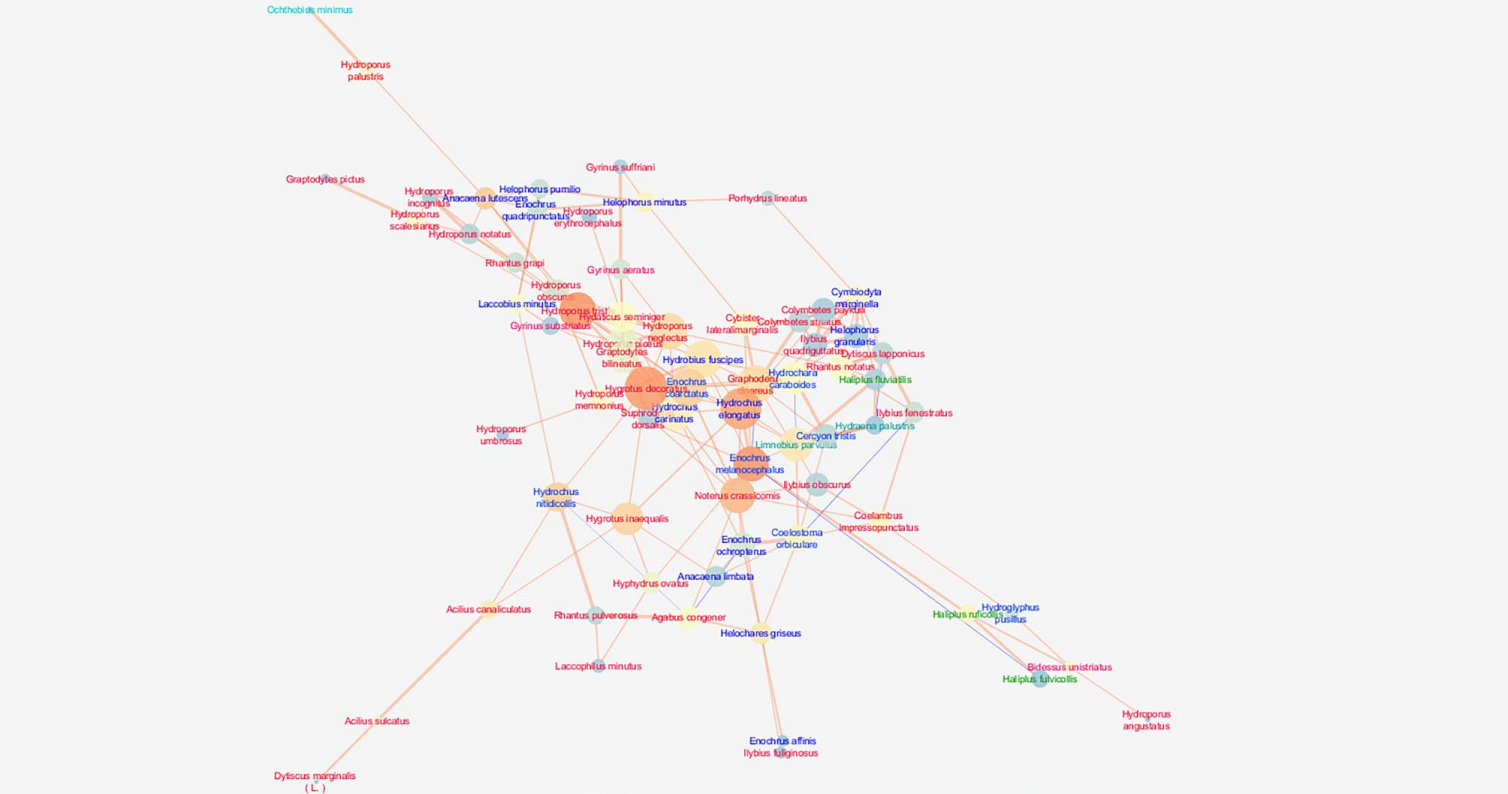
Network graph analysis of the interactions between beetles species in the Masurian Lakeland with node closeness centrality (NCC), node betweenness centrality (NBC) and correlation coefficient (R). Refer to the legend and explanations in Fig. 3 (Kashubian Lakeland). The graph was generated using the Metscape application in the Cytoscape 3.7.2 package, https://cytoscape.org.

The network of interspecific interactions observed in the Masurian Lakeland (Masurian Region) was characterised by a moderate cohesion metric (clustering coefficient = 0.277) and density metric (0.070), while the centrality coefficient (centralisation) was the highest (0.107). At the same time, the highest values for the average number of neighbours (4.829) per species (node) and the number of shortest paths (4830) we observed in this network, as well as moderate values for the characteristic path length (3.332). This network also has the greatest fragmentation, as shown by the highest value of the network heterogeneity parameter (4.09) (Fig. 4; Table 3).

**Figure 5:**
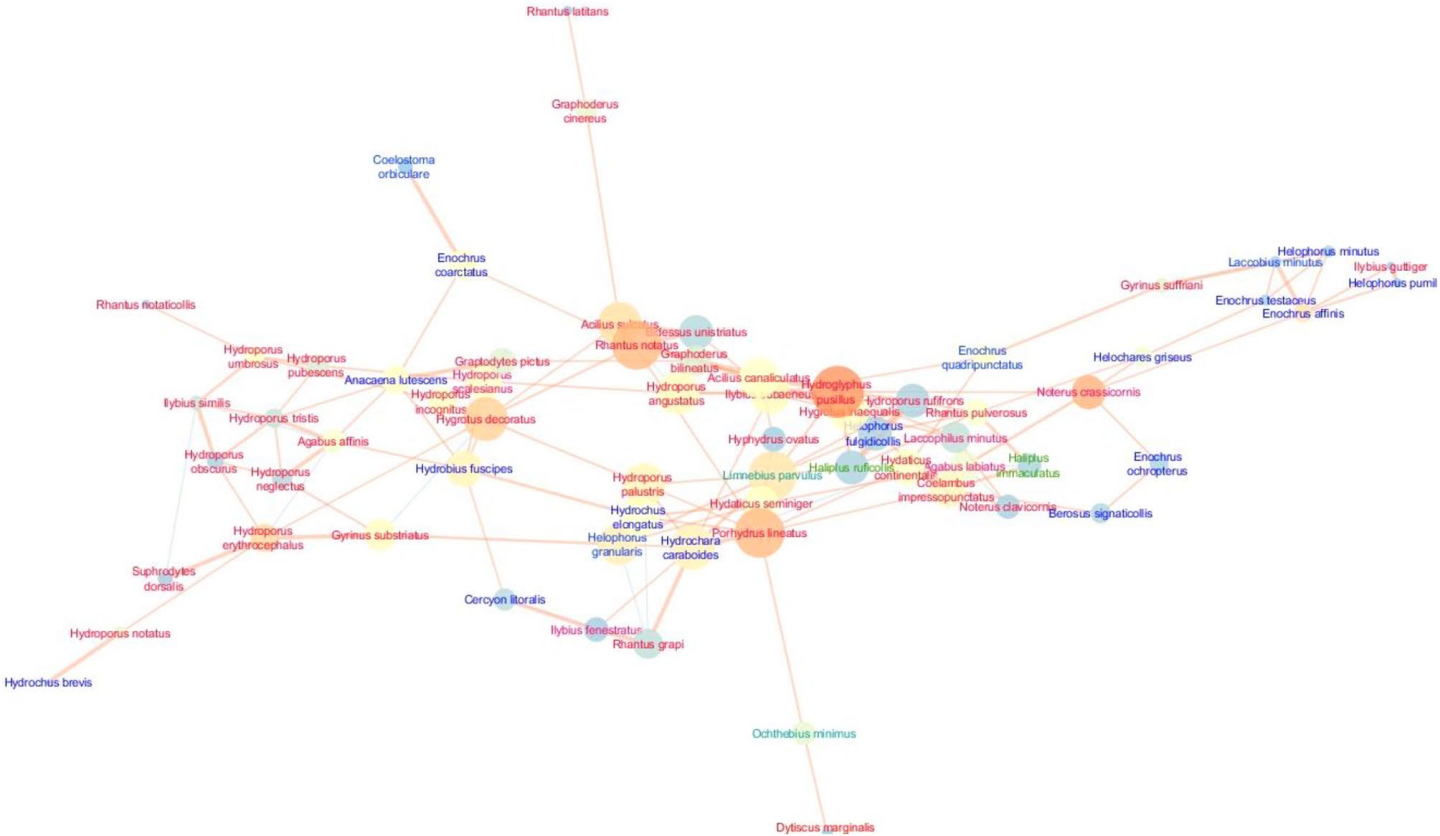
Network graph analysis of the interactions between beetles species in the Suwalki Lakeland with node closeness centrality (NCC), node betweenness centrality (NBC) and correlation coefficient (R). Refer to the legend and explanations in Fig. 3 (Kashubian Lakeland). The graph was generated using the Metscape application in the Cytoscape 3.7.2 package, https://cytoscape.org.

The lowest cohesion and density characterise the network in Suwalki Lakeland. This is confirmed by the values for clustering (0.234) and density (0.062) and centralisation (0.077). The network also had the lowest value for the average number of neighbours (4.829) per species (node) and a moderate number of shortest paths (4422). At the same time, the presence of taxa communicating with the largest number of species was the highest among the three analysed networks, as shown by the value of the characteristic path length (3.332) (Fig. 5; Table 3).

### Interspecific relationships in water beetles networks

An important measure of interspecific relationships is node degree centrality (NDC), which describes the number of direct connections with a given taxon (node) (Table 4). We found the highest NDC values (12) in the Mazurian region for *Hygrotus decoratus* (12), *Hydrochus elongatus* (11) and *Hydroporus neglectus* (10). In the other two regions, the maximum NDC values were lower. In the Suwalki Lakeland, they were observed for *Hydroglyphus pusillus* (9) as well as *Porhydrus lineatus* and *Noterus crassicornis* (8) and in the Kashubian Lakeland – for *Hydroporus angustatus* and *Hydroporus tristis* (9) as well as for *Anacaena lutescens*, *Hydroporus angustatus* and *Enochrus ochropterus* (8) (Table 4). In most cases, they were the most numerous species in each region (Table 1).

**Table 4:** Water beetles species with the highest net attribute.

	Kashubian Lakeland			Mazurian Lakeland			Suwalki Lakeland		
	NCC	NBC	NDC	NCC	NBC	NDC	NCC	NBC	NDC
	0.19–0.40	0.0–0.31	1–8	0.18–0. 42	0.0–0.14	1–12	0.18–0.37	0.0–0.20	1–9
Hydrobius fuscipes*	0.28	0.18	4						
*Ilybius ater*		0.18							
*Helochares griseus*	0.40	0.31	8						
*Enochrus ochropterus*	0.31	0.10	7						
Anacaena lutescens*	0.38	0.18	7	0.31	0.09	7	0.28	0.04	5
*Rhantus bistriatus*	0.36	0.04	7						
Hydroporus palustris*	0.36	0.11	7						
*Hydroporus angustatus*	0.35	0.04	8						
*Limnebius parvulus*	0.36	0.04	7						
Hydroporus tristis*	0.36	0.12	8						
*Hygrotus decoratus*				0.42	0.14	12			
*Hydrochus elongatus*				0.41	0.13	11			
*Hydroporus neglectus*				0.39	0.07	10			
*Cymbiodyta marginella*				0.31	0.03	9			
*Enochrus melanocephalus*				0.38	0.14	9			
Noterus crassicornis*	0.32	0.06	3	0.38	0.11	9	0.28	0.04	8
*Hydroglyphus pusillus*							0.37	0.20	9
*Porhydrus lineatus*							0.37	0.15	8
*Hydroporus erythrocephalus*							0.28	0.10	6
*Rhantus notatus*							0.36	0.15	7
Enochrus affinis*	0.32	0.06	3	0.23	0	1	0.24	0.09	5

*NCC* node closeness centrality, *NBC* node betweenness centrality, *NDC* node degree centrality.

*The most numerous species.

The parameter that indicates the importance of species in a network in terms of their influence on other species is the node closeness centrality (NCC) (Table 4). We found the highest NCC values (from median to maximum) for the network in the Masurian Lakeland (0.18–0.42), where the highest proportion of important species was also found (96%). The most important species was *Hygrotus decoratus* (0.42), followed by *Hydrochus elongatus* (0.41), *Hydroporus neglectus* (0.39), *Enochrus melanocephalus* (0.38) and *Noterus crassicornis* (0.38). We found the lowest range of this centrality attribute (0.19–0.40) for the Kashubian Lakeland, where almost half of the species were ranked as most important. The most important species were: *Helochares griseus* (0.40), *Anacaena lutescens* (0.38), *Hydroporus tristis*, *Hydroporus palustris*, *Hydroporus angustatus*, *Rhantus bistriatus* and *Limnebius parvulus* (0.36). The range of NCC values for the Suwalki Lakeland network was lower (0.18–0.37). Here, over 705 species were distinguished by the highest value of the centrality attribute. The most important species were: *Hydroglyphus pusillus*, *Porhydrus lineatus* (0.37), *Rhantus notatus* (0.36), *Hygrotus decoratus* (0.36), *Anacaena lutescens*, *Noterus crassicornis* (0.28) and *Enochrus affinis* (0.24) (Table 4).

The contribution of individual species to the cohesion of an entire network can be measured with the metric of node betweenness centrality (NBC). The highest value (0.31) was recorded in the Kashubian network for *Helochares griseus* (0.31), followed by *Anacaena lutescens* (0.18), *Hydrobius fuscipes* (0.18), *Ilybius ater* (0.14) and *Hydroporus tristis* (0.12) (Table 4). This attribute favours the species (nodes) that connect to clusters (subnetworks) consisting of other species. As a result, the network is less coherent and more fragmented. Species (nodes) that communicate with other clusters of the network play a more important role than those that are within the sub–networks. In the network of interspecific interactions developed for the Masurian lakes, we found the highest NBC values for *Hydroporus tristis* (0.15), *Hygrotus decoratus*, *Enochrus melanocephalus* (0.14), *Hydrochus elongatus* (0.13) and *Noterus crassicornis* (0.11) (Table 4). In Suwalki Lakeland (where we recorded significantly lower NBC values than in the other regions), *Hydroglyphus pusillus* (0.20), *Porhydrus lineatus*, *Rhantus notatus* (0.15) and *Hygrotus decoratus* (0.12) made the greatest contribution to network cohesion. This network also had the lowest number of species unimportant for network cohesion (with the lowest NBC values) (Table 4).

The correlation coefficient (r) and edge betweenness centrality (EBC) are important metrics for describing relationships between nodes (species). In the Kashubian region, EBC values ranged from 2 – 305.93 (Table 5). The lowest values occurred 11 times, including such pairs as: *Helophorus granularis* and *Helophorus griseus*, *Agabus affinis* and *Anacaena lutescens*, and *Gyrinus aeratus* and *Gyrinus paykuli*. The correlation for the species pairs mentioned was complete (r = 1). Values of EBC > 200 were again found only for relationships between *Anacaena lutescens* and *Helochares griseus*, *Hyphydrus ovatus* and *Helochares griseus*, *Ilybius ater* and *Hydrobius fuscipes* and *Ilybius ater* and *Graptodytes pictus*. We found no statistically significant negative relationships in the networks of interspecific relationships (Table 5).

**Table 5:** Highest values of edge (relations) betweenness centrality (EBC) for beetles species and connections among them.

Pair of species	r	EBC
Kashubian Lakeland		
* Anacaena lutescens* &* Helochares griseus*	0.29	305.93
* Ilybius ater* &* Hydrobius fuscipes*	0.72	272.65
* Hyphydrus ovatus* &* Helochares griseus*	0.30	260.45
* Hydroporus erythrocephalus* &* Hydrobius fuscipes*	0.43	182.77
* Graptodytes pictus* &* Ilybius ater*	0.30	257.83
Mazurian Lakeland		
* Haliplus ruficollis* &* Enochrus melanocephalus*	0.79	329.19
* Hydroporus scalesianus* &* Hydroporus tristis*	0.24	251.48
* Hydroporus tristis* &* Enochrus coarctatus*	0.41	214.29
* Hydroporus tristis* &* Anacaena lutescens*	0.54	323.67
* Acilius canaliculatus & Acilius sulcatus*	0.94	272
Suwalki Lakeland		
* Noterus crassicornis* &* Hydroglyphus pusillus*	0.34	620.99
* Noterus crassicornis* &* Enochrus affinis*	0.29	481.17
* Hydroporus erythrocephalus* &* Hygrotus decoratus*	0.23	372.81
* Hygrotus decoratus* &* Rhantus notatus*	0.47	334.63
* Gyrinus substriatus* &* Helophorus granularis*	0.64	286.11

In the Masurian Lakeland, the range of EBC values was wider (2–329.19) (Table 5). The lowest values occurred 5 times, while the values > 100 were found in 65 cases (including 32 statistically significant cases, p < 0.05). Among the species pairs for which the shortest communication paths (EBC) were determined, 5 pairs were distinguished: *Enochrus quadripunctatus* and *Helophorus pumilio*, *Colymbetes paykuli* and *Ilybius quadripunctatus*, *Colymbetes paykuli* and *Helophorus granularis*, *Ilybius quadripunctatus* and *Helophorus granularis*, and *Graptodytes bilineatus* and *Hydroporus piceus* (r = 1). For the pairs *Haliplus ruficollis* and *Enochrus melanocephalus*, *Hydroporus tristis* and *Anacaena lutescens*, *Hydroporus scalesianus* and *Hydroporus tristis*, values of EBC > 200 were again found. Statistically significant negative correlations were also found between the following pairs: *Agabus congener* and *Hydrochus nitidicollis* (r = – 0.65), *Hydrochara caraboides* and *Limnebius parvulus* (r = – 0.46) and *Dytiscus lapponicus* and *Hydraena palustris* (r = – 0.27 (Table 5).

In Suwalki Lakeland, the range of EBC values was the widest (2 – 620.99) (Table 5). We recorded the lowest EBC values 5 times (p < 0.05) for the pairs: *Haliplus ruficollis* and *Hydroporus rufifrons*, *Haliplus ruficollis* and *Helophorus fulgidicollis*, *Hydroporus rufifrons* and *Helophorus fulgidicollis*, *Ilybius guttiger* and *Helophorus pumilio*, and *Helophorus minutus* and *Laccobius minutus*. EBC > 100 values were found in 67 cases (of which 45 were statistically significant, p < 0.05), of which the highest values were for the pairs *Hygrotus decoratus* and *Rhantus notatus* (EBC = 334.63), *Hydroporus erythrocephalus and Hygrotus decoratus* (372.81), *Noterus crassicornis* and *Enochrus affinis* (481.17), *Rhantus notatus* and *Hydroglyphus pusillus* (450.27) and *Noterus crassicornis* and *Hydroglyphus pusillus* (620.99). These long communication paths lead to a fragmentation of the network. Negative correlations occurred in the pairs: *Rhantus grapi* and *Coelostoma orbiculare* (r = – 0.63), *Rhantus grapi* and *Hydroporus palustris* (r = – 0.28), *Gyrinus suffriani* and *Helophorus minutus* (r = – 0.29), *Hydaticus continentalis* and *Haliplus immaculatus* (r = – 0.24) (Table 5).

### Influence of biotic and environmental factors on species attributes in water beetles networks

**Figure 6:**
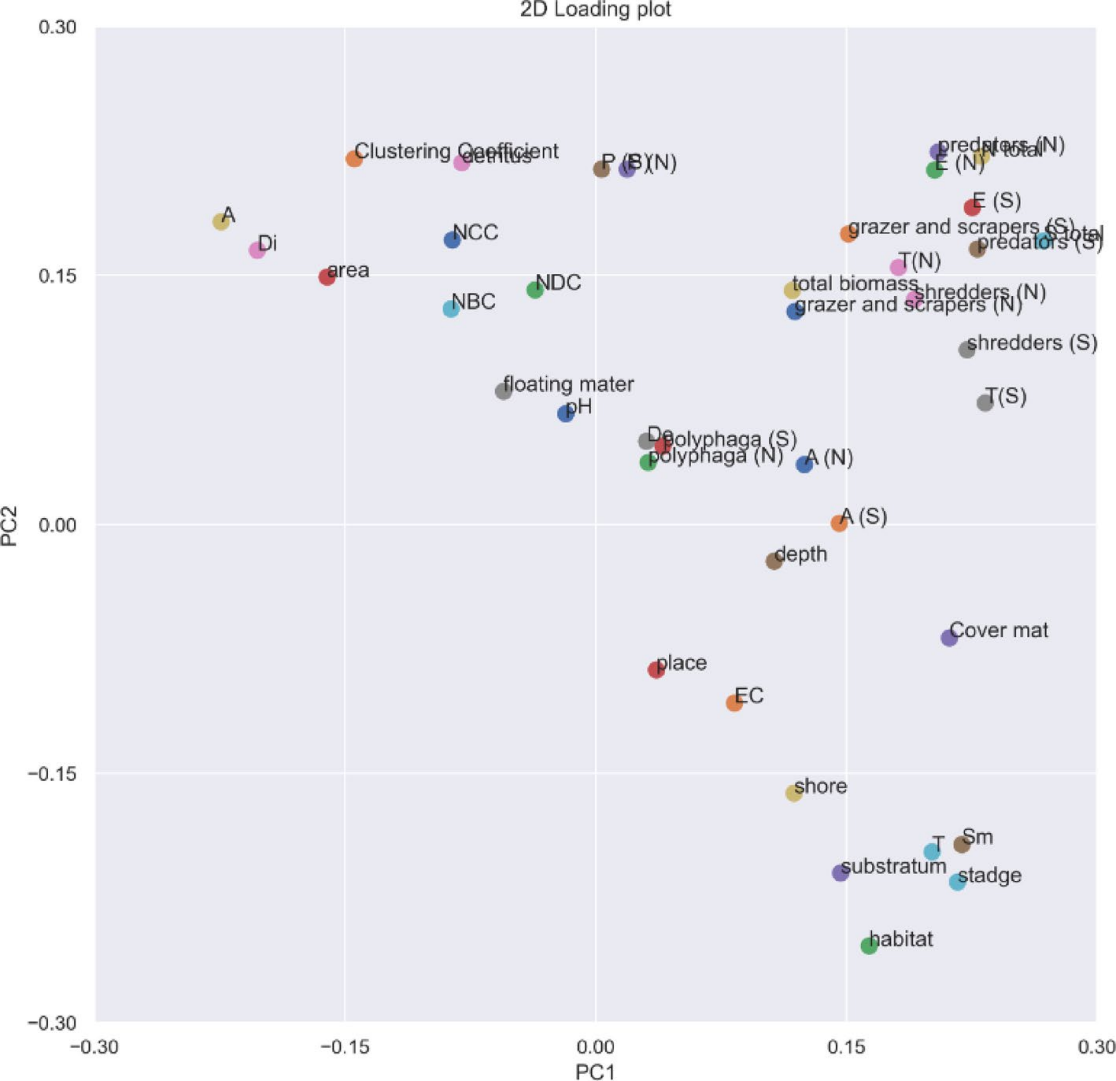
Principal component analysis (PCA) order diagram of ecological groups, functional groups, environmental variables and species network attributes in the samples along the first and second PCA axes. ‘N’ – abundance, ‘S’ – number of species, ‘NCC’ – (node closeness centrality), ‘NBC’ – (node betweenness centrality), ‘NDC’ – (node degree centrality) and ‘correlation coefficient’. The values for the other variables analysed are listed in Table 6. The plot was created in the programming language Python 3.9 using the libraries “Matplotlib” and “Scikit–Learn” (module “PCA”), documentation: https://scikit-learn.org/stable/modules/generated/sklearn.decomposition.PCA.html.

The results of the PCA show that both coding axes (PC1 and PC2) together explain 40% of the total variance of the variables (Fig. 6; Table 6). With the PC1 axis, the following variables showed the strongest positive correlations: ‘abundance of tyrphophiles ‘T(N)’, number of species of tyrphophiles ‘T(S)’, total abundance ‘N total’, total number of species ‘S total’, ‘predators (N)’, ‘predators (S)’, ‘shredders (N)’, ‘shredders (S)’, ‘area’, ‘Cover mat’, *Sphagnum* mat ‘Sm’, and negatives – ‘Depth’, habitat with diffuse macrophytes ‘Di’, habitat with dense macrophytes ‘De’, psammolittoral zone ‘A’, again with axis PC2: ‘P (N)’, abundance of lake and river species, ‘P (S)’, ‘T(N)’, ‘T(S)’, ‘N total’, ‘S total’, ‘grazer and scrapers (N)’, ‘grazer and scrapers (S)’, ‘total biomass’, ‘NBC’, ‘NCC’, ‘NDC’, ‘Clustering Coefficient’, ‘habitat’, ‘place’, ‘substratum’, ‘depth’, ‘detritus’, ‘floating matter’, ‘shore’ and ‘stage’.

**Table 6:** The characteristics of studied dystrophic lakes within three regions in Poland.

Parameters	Kashubian Lakeland	Mazurian Lakeland	Suwalki Lakeland
Morphometric			
Number of lakes	7	12	6
Area (ha) – area of lakes	0.4–17.5	0.4–4.24	0.45–8.9
Cover mat (%)	2–14.0	3–70	31–75
Contribution in the littoral zone (%)			
*Sphagnum* mat (Sm)	0.1–0.8	0.5–0.9	0.8–0.9
Diffuse macrophyte (Di)	0.1–0.7	0.1–0.2	0.1–0.2
Dense macrophyte (De)	0.0–0.1	0.0–0.4	0.0–0.0
Arenal zone (psammolittoral) (A)	0.0–0.3	0.0–0.1	0.0–0.1
Trophic type	O–P	M–P	P
Stage	1 – young, 2 – mature, 3 – old		
Floating matter	1 – none, 2 – present		
Sampling depth (cm)	10–50		
Shore	1 – flat, 2 – steep		
Detritus	1 – none, 2 – scarce and fine, 3 – abundant and fine, 4 – abundant and coarse		
Habitat	1 – arenal, 2 – diffuse macrophytes, 3 – dense macrophytes, 4 – *Sphagnum* mat		
Soil substrate	1 – sand, 2 – sand and mud, 3 – *Sphagnum*		
Place	1 – ecotone, 2 – pockets and ponds within the *Sphagnum* mat 3 – pressed *Sphagnum* mat		
T – trophic type	1 – oligohumic, 2 – oligo–mesohumic, 3 – mesohumic, 4 – meso–polihumic and polihumic lake		
Hydrochemical parameters			
Temperature (°C)	17.3 ± 6.1		
O2 (%) – oxygen saturation content	85.0 ± 10.0		
pH	5.43 ± 0.68		
HDI – hydrochemical dystrophy index	142.2 ± 21.0		
EC (mScm/1) – electrical conductivity	5.29 ± 0.82		

We found the highest correlations between ‘predators (N)’ and ‘N total’ (r = 0.93), ‘predators (S)’ and ‘S total’ (r = 0.86), ‘E (N)’ and ‘N total’ (r = 0.87) and ‘E (S)’ and ‘S total’ (r = 0.88). Significant correlations were found between lake maturity stage ‘T’ and ‘cover mat’ (r = 0.56), proportion of *Sphagnum* mat ‘Sm’ (r = 0.83) with ‘habitat’ (r = 0,64) and the ‘substratum’ (r = 0.48) and the proportion of the psammolittoral ‘A’ (r = – 0.83) with the habitat with diffuse plants ‘Di’ (r = – 0.76) and the ‘area’ (r = – 0.73) were found.

Significant correlations were also found between: ‘area’ and ‘A’ (r = 0.56), 'Di' (r = 0.58), ‘cover mat’ (r = – 0.62) and 'Sm' (r = – 0.42), ‘T (S)’ (r = 0.40). Significant positive correlations were found between ‘Sm’ and ‘N total’ (r = 0.32), 'S total' (0.47) and ‘total biomass’ (r = 0.31). Noteworthy are the correlations between 'Sm' and ‘N total’ (r = 0.32), ‘S total’ (r = 0.47), tyrphophiles ‘T (S)’ (r = 0.36) and ‘total biomass’ (r = 0.31) and between ‘detritus’ and lake and river species ‘P (N)’ (r = 0.37) and ‘P (S)’ (r = 0.44) and between different ecological elements, e.g. tyrphophiles and argilophiles – for abundance (N): r = 0.15, while for number of species (S): r = 0.25.

We found significant correlations between species network attributes and environmental factors. Negative correlations were found between cover mat and ‘NBC’ (r = – 0.36), ‘NCC’ (r = – 0.34), ‘Clustering Coefficient’ (r = – 0.33) and ‘NDC’ (r = – 0.22). The correlations between ‘Sm’ and the specified network attributes were: (r = – 0.37, r = – 0.24, r = – 0.38, r = – 0.10). Lower correlations were found between ‘depth’ and the subsequent network attributes (r = – 0.28, r = – 0.35, r = – 0.20, r = – 0.37) and for 'T' – (r = – 0.26, r = – 0.25, r = – 0.38, r = – 0.12). Positive correlations were found between 'Di' and the distinguished network attributes (r = 0.32, r = 0.17, r = 0.30, r = 0.03), ‘De’(r = 0.25, r = 0.33, r = 0.10, r = 0.40), ‘A’ (r = 0.24, r = 0.17, r = 0.43, r = 0.01), ‘area’ (r = 0.21, r = 0.21, r = 0.33, r = 0.07). All described attributes of the species network showed slight correlations with ‘N total’ and ‘S total’.

## Discussion

### The influence of environmental factors on the structure of the beetle fauna

The abundance of scientific references indicates that dystrophic, humic lakes create unfavourable habitat conditions for aquatic organisms, resulting in species and quantity poverty of fauna^34,35,40–42^. Our results, which are part of a broader study on the ecology of beetles in dystrophic lakes^4,6^, do not confirm the above opinion. The species richness of beetles (104 species) in the lakes selected for our study accounts for a quarter of all species reported for Poland^43^.

We performed additional analyses carried out in our study using an innovative tool^21,23^ such as the theory of graphs to analyse a structure, which allowed us to make a very informative assessment of the interspecific interactions in the networks of relationships in assemblages of beetles in lakes. The results of these analyses also show clear regional differences in the beetle fauna of the lakes studied.

The reasons for these differences lie in the nature of the lakes in the three regions (their general physiognomy, especially the spatial structure of the littoral zone), which undoubtedly results from their development and relative ecological age. The lakes in the Suwalki Lakeland are ecologically older than the Masurian lakes and even older than the lakes in the Kashubian (Pomeranian) Lakeland, where the last, Pomeranian phase of the last Pleistocene glaciation, known as the North Polish Glaciation (also known as the Vistula Glaciation), had a wider range and ended later^33,44,45^.

The ageing of dystrophic humic lakes is shown by the successive disappearance of the psammolittoral zone and the reduction of the water surface of the lake due to the sliding of the *Sphagnum* mat^6,36,46^. This is confirmed by the results of our investigations, which show correlations between the successional stage of the lake and the degree of development of the *Sphagnum* mat, the cover mat (positive correlations), as well as the surface area of the reservoir, the proportion of other habitats in the littoral of the lakes, such as sandy bottom habitat, without plants or habitats with diffuse macrophytes (negative correlation) (Fig. 6). *Sphagnum* mat covered 70–80% of the water surface in the lakes of the Suwalki Lakeland, while in the Kashubian lakes it represents only a small part of the phytolittoral zone. As a result, younger lakes have a more diverse shoreline (greater diversity of habitats), in contrast to older lakes where this spatial structure is impoverished but the fractal dimension of the lake increases^6,47,48^. Fractal structure plays an important role in shaping trophic relationships between species in the aquatic environment^3,6,49,50^. A compact *Sphagnum* mat in lakes creates a complex spatial structure that increases the surface area that can be inhabited by a very rich and diverse fauna of invertebrates, but eliminates species with larger body size, which reduces predation pressure^6,51^. This regularity is also confirmed by our studies showing positive correlations between the *Sphagnum* mat and the abundance of beetles, especially small argilophiles and tyrphophiles (Fig. 6).

Many hydrobiologists point out that the spatial structure of aquatic ecosystems, including lakes dominated by plant habitats, is much more important for the occurrence of macroinvertebrates than the physico–chemical properties of the water^1,2^. Habitats with macrophytes cause fragmentation of space, transforming it into a much more complex area that is more accessible for colonisation^51–53^. At the same time, they create suitable places to hide from predators (including fish), search for food and lay eggs^52,53^. Plants as such are also a rich source of food in the form of dead organic matter and periphyton growing on plants^51^. This is also confirmed by the increase in the value of the correlation coefficient between the degree of overgrowth of the habitat and the availability of detritus and the abundance of beetle communities (Fig. 6). The development of macrophytes and the extent to which the littoral is covered with plants are a measure of succession in a lake, and the succession of a lake in turn leads to directional changes in the fauna of the organisms living in that lake. It leads to a restructuring of species composition and a change in ecological structure^4–6^. This process is particularly evident in organisms with good bioindicator properties. Aquatic beetles are generally a group of species with a broad ecological tolerance, which is why – as numerous references confirm – the fauna of aquatic environments is dominated by eurytopes^54–56^. The greatest importance of eurytopes for both the abundance of the collected material and the species diversity is also confirmed in our study (Fig. 1).

The specific character of a given ecosystem depends on more specialised elements^7–11,54,55,57^. Among the beetles of the lakes studied, the most typical, i.e. lake and river species, include *Ilybius fenestratus*, *Ilybius fuliginosus*, *Gyrinus aeratus*, *Gyrinus marinus*, *Porhydrus lineatus* and *Haliplus fluviatilis*^8,59^ (Table S1). This group of species prefers clean, well–aerated waters and settles in places that are only sparsely covered with macrophytes. This explains why lake and river species are significantly more numerous in the younger Kashubian lakes, while they are least numerous in the Suwalki Lakeland, which is confirmed by the results of a study on harmonic lakes^5^. The most typical element for peat waters, i.e. tyrphophiles (with the most frequent *Anacaena lutescens*, *Hydroporus tristis* and *Enochrus affinis*), were most numerous in the Suwalki Lakeland and the Masurian Lakeland, while they were somewhat less frequent in the Kashubian Lakeland. It is worth mentioning that argilophiles: *Hydrogyphus pusillus*, *Laccobius minutus*, *Helophorus granularis*, *Helophorus minutus*, *Helophorus fulgidicollis* and *Helophorus pumilio*, were more numerous in the Suwałki region, while they made up only a small proportion in the lakes of the other two regions (Table S1). Our PCA results (Fig. 6) confirm the observations that species preferring waters with higher mineralisation often have an affinity for peat bog waters and are relatively more numerous among acidophilic tyrphophilous species^58,59^.

Water beetles are among the organisms that tap various food resources^14–16,59^. Species from the families Dytiscidae, Noteridae and Gyrinidae are actively hunting predators, Hydrophilidae are mainly shredders and feed on macromolecular organic material, although *Hydrophilus* sp. larvae are also predators, Hydraenidae (*Limnebius parvulus*, *Ochthebius minimus* and *Hydraena palustris*) are grazers and scrapers feeding on particulate organic matter while Haliplidae are polyphagous and feed mainly on plant food but also on small detritus^18,19^. The most numerous beetles in the beetle fauna of aquatic habitats are usually Dytiscidae and Hydrophilidae. Many publications indicate that the quantitative prevalence of predatory beetles testifies to low eutrophication and clean waters, while the eutrophication of waters is indicated by the increasing number of saprophagous detritus–feeding beetles^55^. This study supports the above observation as we found the highest abundance and species richness among predatory beetles in lakes of all three regions. This could indicate that dystrophic waters are poor in organic matter. The question arises how it is possible that so many predators can coexist in lakes with a low number of potential prey (Table 1, Table S1).

Some hydrobiologists^14,16,17,60–62^ emphasise in their studies that predatory beetles feed on quite different foods depending on the food supply and body size (of both the predator and its prey). The beetle feeds mainly on other insects, especially Ephemeroptera, Chironomidae, but also on other Diptera, Heteroptera, ground beetles that happen to fall into the water, frog eggs, fish fry, adult beetles and their larvae, even of the same species. The diet can be supplemented by fragments of aquatic plants^14,60–62^. Some authors point out the positive correlation between the body size of a predator and its potential prey^60^. All this information explains very well the nature of the graphs illustrating the networks of relationships between beetles in the regions studied. One indicator of the interspecific relationships in the network studied is the biomass flow according to Fath et al.^25^ as also used in research on zooplankton^21–23^.

### Interspecific relationships in water beetles networks

The network of relationships in the Kashubian Lake District, despite the lowest number of species (nodes), is characterised by the highest number of interactions compared to the other analysed regions, affecting clustering and network density (Fig. 3), which is indicated by negative correlations between successional stage and species network attributes (NDC, NCC, NBC) (Fig. 6). Although the low number of very high correlations between species (shortest paths) causes the lower density of the network and the highest value of network heterogeneity compared to the Masurian Lakes. This network is moderately centralised, which is due to the fact that species important for the network correlate rather weakly with other species forming coherent clusters (sub–networks). This is the case, for example, with *Anacaena lutescens*, which shows strong correlations with the species: *Hydroporus tristis*, *Hydroporus angustatus*, *Hydroporus palustris*, and through its relationship with *Hydroporus tristis* it also correlates with *Hydroporus neglectus* and forms a cluster with *Hydroporus scalesianus* and *Coelostoma orbiculare*. The mentioned relationships between species in the central part of the network concern tyrphophiles with small body size, which are mainly found in habitats with *Sphagnum* mats, which is also reported by Bloechl et al.^52^. This finding points to the key role of body size for the structure of a food web and especially for its stability^20,26,27^.

The network also contains homogeneous clusters consisting of strongly correlating argilophilous species, such as: *Laccobius minutus*, *Helophorus griseus*, *Helophorus granularis*, which in turn are connected to the cluster of eurytopes: *Enochrus ochropterus*, *Enochrus coarctatus* and *Hydrochus carinatus*. They all form a cluster of saprophagous beetles (shredders) that occur in the shallower littoral of the lakes, on the sandy lake bottom, in places with accumulated organic material, which is confirmed by studies by Biesiadka^54^ and Kordylas^55^. In the network, there is an obvious tendency for peripheral settlement of predatory species with larger bodies, which are usually better swimmers and tend to emerge deeper in the lake waters in places sparsely covered with macrophytes (e.g. lake and river species, such as: *Ilybius fenestratus* and *Ilybius fuliginosus*) or are more densely vegetated (e.g. eurytopes: *Dytiscus marginalis* and *Acilius sulcatus*, or tyrphophiles: *Acilius canaliculatus* and *Colymbetes fuscus*), but also on the open water surface of a lake (e.g. *Gyrinus aeratus* and *Gyrinus paykuli*). Such a structure of the network, a large number of connections, its rarity and the presence of clear clusters are undoubtedly the result of a very diverse littoral zone of the lakes and a small number of scattered habitats with *Sphagnum* mat that allow the dispersal of species.

The Mazurian network is characterised by the highest centralisation (Fig. 4). There are many very strong correlations between species. The species that are most important for the network (the highest NCC) and play the most important role for the cohesion of the network (the NBC) are typically small–sized tyrphophiles (*Hygrotus decoratus*, *Hydroporus incognitus*, *Hydroporus neglectus*, *Enochrus melocephalus*, *Enochrus coarctatus*) located in the centre of the network. They show strong correlations with other species, both tyrphophiles and eurybionts. This assemblage (this fragment of the network) reflects the relationships between the beetles inhabiting the *Sphagnum* mat. The periphery of the network, as in the Kashubian network, consists of species inhabiting other habitats not occupied by a *Sphagnum* mat. These include lake and river species (*Gyrinus aeratus*, *Porhydrus lineatus*, *Haliplus fluviatilis*, *Ilybius fenestratus*, *Ilybius fuliginosus*) in less vegetated areas, eurytopes (*Dytiscus marginalis*, *Acilius sulcatus*, *Hydrochara caraboides*) in densely vegetated areas and argilophiles (*Laccobius minutus*, *Helophorus pumilio*, *Helophorus minutus*, *Helophorus granularis* and *Hydroglyphus pusillus*) on the sandy bottom without macrophytes^8^. The nature of this network is undoubtedly explained by the presence of different habitats in the littoral of the lakes studied, but above all by the wider and more compact *Sphagnum* mat found here compared to the Kashubian network, which led to a high correlation of these variables with the dominant species (Fig. 6).

We identified the least dense and most heterogeneous network in the Suwalki Lakeland (Fig. 5). This is due to the fact that the species important for the network (*Hygrotus decoratus* and *Hydroglyphus pusillus*), which have a high edge betweenness centrality (EBC) among themselves, have very weak relationships (Table 5). This was a consequence of the different ecological adaptations and occupied habitats. This is confirmed by the study of Allesina and Pascual^20^ who suggest that stronger interactions between species, i.e. shorter predator–prey cycles, stabilise a network. All species important to the network scored relatively low NCC values, leading to a dilution of the network of relationships. This is confirmed by the observations of other hydrobiologists who, for example, studied interdependencies in zooplankton^21,23^. At the same time, higher complexity of a network means its lower stability, which Klecka^13^ underlines.

The network divides into two parts (Fig. 5). The left side consists of a fairly homogeneous assemblage of predatory tyrphophiles, usually of small body size, concentrated on *Hygrotus decoratus* and *Anacaena lutescens*, which are usually numerous on the pressed *Sphagnum* mat. They are accompanied by a few poorly swimming eurytopes (*Hydrobius fuscipes*, *Hydrochus brevis*, *Cercyon litoralis*). The right side of the network consists of a collection of many different ecological and functional elements. The periphery of the entire web of relationships is occupied by large, highly mobile predators, which mostly found at greater depths in the lakes (at some distance from the edge of the *Sphagnum* mat), in the open surface waters of the lakes. This is confirmed by the observations of other scientists^4,12,13,26,27^ who claimed that large predators, e.g. *Dytiscus* sp, are more active in a less complex environment, especially when not threatened by other large predators, e.g. fish^13,26,27^.

Separate clusters in networks consist of small shredders, which are most numerous in the marginal areas of the *Sphagnum* mat covered with scattered macrophytes. Here, too, there were only a few eurytopic polyphages. This situation is consistent with the results of another study^51^ which found that macrophytes are an important component of the diet of herbivores, while dead plant fragments are available as organic material for saprophages. The specificity of this network is explained by the emergence of a new potential habitat in mature polyhumic lakes in the form of bays or reservoirs in broken *Sphagnum* mat, where large predatory beetles like to occur and which are avoided by small organisms. This is confirmed by the negative correlation between ‘habitat’ and network attributes of these species (NDC, NCC, NBC) (Fig. 6). The great diversity of beetle communities in mature lakes is due to the diversity of available habitats. In shallower *Sphagnum* mat habitats, the number of potential connections is greater (negative correlations of attributes with ‘depth’). This habitat creates very good conditions for the occurrence of small organisms, but is a strong barrier for large organisms, which reduces the number of potential connections in the polyhumus lakes of the Suwałki Lakeland.

In all three networks, positive correlations between beetle species predominate, proving the coexistence of most species. This is certainly a consequence of sharing an ecological niche with abundant food resources, which reduces competition. This is favoured by the more complex spatial structure of the habitat with a high fractal dimension, which makes it inaccessible to potential large predators^3,4,26,27,49,57^. The *Sphagnum* mat is the habitat with the richest and most diverse species fauna of small–bodied beetles, which is also confirmed by other researchers^4,63^.

We observed significant negative correlations only in the Mazurian network (including pairs: *Agabus congener* and *Hydrochus nitidicollis*, *Dytiscus lapponicus* and *Hydraena palustris*, and *Hydrochara caraboides* and *Limnebius parvulus*) and in the Suwalki Lakeland (including pairs: *Hydaticus continentalis* and *Haliplus immaculatus*, *Rhanthus grapi* and *Cercyon litoralis*, *Helophorus granularis* and *Hydrochus elongatus*, *Agabus labiatus* and *Noterus clavicornis*). All interactions are examples of predation on prey from lower trophic levels by larger predators (shredders, grazers and scapers, and polyphages). Only the interaction between the predators *Agabus labiatus* and *Noterus clavicornis* shows mutual elimination through competition for resources or devouring species of similar body size, which is confirmed by a study by Frelik^14^.

## Conclusions

In our study, we analysed the structures of beetle communities inhabiting small dystrophic lakes in three regionally different lake landscapes. For this purpose, we created three network models that we subjected to graph network analysis. This method was also used to determine the importance of specific species in the networks as well as the interspecific relationships. The measurement parameter in our analyses was the biomass of the individual species that make up the food webs, as well as their individual characteristics, which allow us to distinguish between ecological elements and functional trophic groups. We verified all four hypotheses presented in the introduction. We have shown that the networks of relationships between species in the three regions studied differ significantly in terms of cohesion, density, centralisation of the network, degree of dilution and heterogeneity, which can be related to the fractal structure and the degree of development of the lakes studied (H1). The networks are dominated by positive mutualistic relationships between the beetle species (H2). It is possible to identify the species that are particularly important for the stability of the network (H3). The networks tend to divide into clusters consisting of species with similar habitat and food preferences (H4). The central places in the network were typically occupied by small tyrphophiles, which were also the most important species for network cohesion and density, while the periphery of the network consisted of clusters with different habitat preferences, including large predators. The values of attributes determining the role of species in community networks were influenced by both biotic and environmental factors. The analysis of graph networks offers a new insight into the mutual relationships between species in beetle communities.

## Material and methods

### Study area

**Figure 7:**
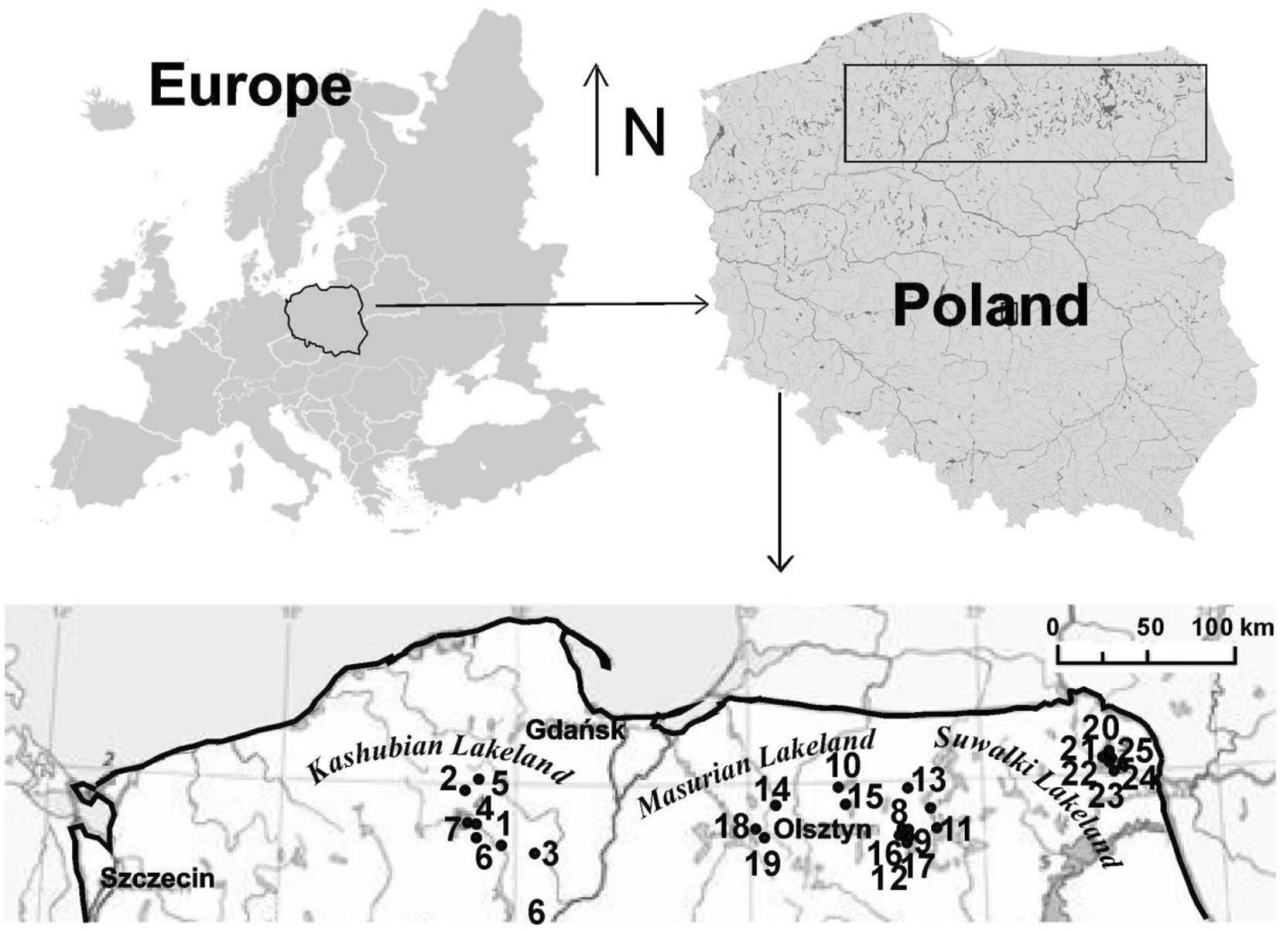
Study area showing the location of the lakes. 1, Długie; 2, Klimontek; 3, Małe Gacno; 4, Małe Łowne; 5, Piecki; 6, Sosnówek; 7, Żabionek; 8, Bobrówko; 9, Borkowskie; 10, Białe; 11, Gryżewskie; 12, Krucze Oko; 13, Kruczy Stawek; 14, Jonkowo; 15, Kociołek; 16, Kruczek Duży; 17, Kruczek Mały; 18, Motylek; 19, Żabie; 20, Suchar Wielki; 21, Suchar1; 22, Suchar 2; 23, Suchar 3; 24, Suchar 4; 25, Suchar 5. The map was generated using the CorelDRAW 9 application in the Corel, package, https://www.corel.com. Modified, see^4–6^.

We studied 25 dystrophic lakes in northern Poland, i.e. in the Kashubian Lakeland (West Pomeranian Lakeland), in the Masurian Lakeland, and in the Suwałki Lakeland (Fig. 7, Table S2). They had different surfaces and were overgrown to varying degrees by *Sphagnum* mat. They represented different stages of succession in a discordant sequence – from oligohumic to polyhumic lakes. Species inhabiting the oligohumic lakes included *Juncus bulbosus, Eleocharis palustris, Phragmites australis, Typha angustifolia, Typha latifolia, Sphagnum* sp., *Lobelia dortmanna, Isoëtes lacustris, Drosera rotundifolia, D. anglica, D. intermedia, Sparganium angustifolium* and *Lycopodiella inundata*. In the polyhumic lakes, the dominant species were *Sphagnum* sp., *Oxycoccus quadripelatus, Andromeda polifonia, Eriophorum vaginatum, Calluna vulgaris, Erica tetralix* and *Empetrum nigrum*.

The lakes were a priori divided into three groups (successional stages) according to the degree of cover by floating *Sphagnum* mat: I – young, oligohumic lakes (0–3% of the lake area covered by *Sphagnum* mat), II – medium, mesohumic lakes (3–30%), III – mature, polyhumic lakes (> 30%) (Table 6; Table S2). For the classification, we used a modification of the scale proposed by Bayley and Prather^64^. We calculated the area of *Sphagnum* mat in a lake in GIS using ArcGIS software (for Desktop 9.3.1., ESRI, Poland). Sites were mapped using data available in WMS format in Geoportal 2.

### Field studies and collecting samples

The studies were conducted from 2002 to 2013 in spring, summer and autumn. A total of 207 samples were taken and subsequently described on the basis of selected environmental parameters (Table 1). In each lake, sampling was carried out during the entire growing season. The study is part of a broader study on the mechanisms of beetle community formation and functioning as a result of succession of lakes of the harmonic and dystrophic series, which was conducted in the period 2002–2014 and included 70 lakes in northern Poland, over 2000 samples and about 30,000 individuals^4–6^. To achieve the objectives of this work, lakes representing different stages of succession were selected in proportion to their actual share in the studied lake areas. The Kashubian Lake District has the highest number of oligohumic lakes, which are not present in the other lake districts, while the Suwałki Lake District has only polyhumic lakes (Table 6). The number of samples taken in the lake depended on the degree of differentiation of the littoral zone. Samples of fauna were collected with a dip net on an area of about 1m2. From the surroundings of the pressed *Sphagnum* mat environment, 10 subsamples were collected with a 0.1 m2 strainer. The sampling sites were selected to represent the greatest possible diversity of littoral habitats and areas of each lake. Four different littoral components (habitats) were identified: (1) *Sphagnum* mat and ecotone zones between land and water: (2) diffuse macrophyte zone, (3) dense macrophyte zone and (4) arenal zone (sandy bottom habitats). We assessed Plant cover using the phytosociological records of Braun–Blanquet^65^. All lakes were characterised in terms of their surface area, the degree of surface cover by *Sphagnum* mat corresponding to the successional stage (1–3) and the percentage of each habitat in the littoral zone (1–4) (Table 6, Table S2).

The characteristics of a given parameter were described with qualitative values, where a rank corresponded to the strength of a given value. The water parameters, i.e. temperature, pH, electrolytic conductivity and saturation content, were measured with a multiparametric sampling probe Elmetron CX – 401 (Elmetron, Poland). The HDI (Hydrochemical Dystrophy Index; Górniak^66^) was also used for the analyses. The values for the analysed variables are listed in Table 6.

### Ecological and statistical analyses

We calculated species diversity using S – number of species, N – number of individuals and D – percentage and H’ – the Shannon–Wiener index. Three functional groups were distinguished in the trophic structure of the beetles: predators (families: Gyrinidae, Dytiscidae and Noteridae), polyphages (Haliplidae) and saprophages: shredders (Hydrophilidae) and scrapers and grazers (Helophoridae and Hydraenidae)^18,19^. Four ecological groups were distinguished: eurytopes, psammophiles, lake and river species (potamophiles) and tyrphophiles, to determine the holistic character of the fauna^54^. The first group includes species that live in small and highly eutrophic waters. Psammophilous species associated with waters with increased mineralisation show a greater preference for vegetation–free environments with sandy bottom surfaces. They also show a preference for acidified waters. Lake and river species are typical of less eutrophic waters, while the tyrphophilous species are characteristic of various polyhumic waters.

To detect significant statistical differences in species diversity, abundance and biomass of lake beetles in the different regions (lake districts), we used the non–parametric Kruskal–Wallis test. We used the same test to detect significant statistical differences in species diversity, abundance and biomass of the different functional and ecological groups in the study regions. Significant results were tested for pairwise comparisons with a post–hoc test for multiple comparisons of mean ranks for all samples. We performed the Kruskal–Wallis test in Statistica, ver. 13.3 (StatSoft, Tulsa, USA).

The relationships between the biocenotic indicators and network attributes of the beetle assemblages and the analysed environmental parameters in the studied lakes were determined using principal component analysis (PCA). Dimensionality reduction was performed in the Python 3.9 programming language using the Scikit–Learn library.

### Graph network analysis

A graph represents a network of connections (edges) between objects (nodes) by evaluating the properties of the whole network as well as the attributes of nodes and edges in relation to their role in the network, expressed by the metrics of centrality^67^. In this work, we carried out graph analysis with the aim of comparing the properties of the relationship network of insect communities in lakes from three lake regions: Kashubian, Masurian and Suwalki Lakelands. It was also applied to analyse the importance of individual species in these networks and the mutual relationships between them. The network analyses were based on the correlations between the biomasses of trophic levels represented by the beetle species^25^.

The analysis of the network for each lakeland, represented by separate databases, was carried out using the Cytoscape software package (http://www.cytoscape.org/), using the module to create a network based on correlations between nodes. In total, we created three network models from three databases. Insect species divided into functional groups were placed in the columns of the databases, while the rows contained the results of biomass measurements of the species for each sampling date. The data were normalised using the online tool CorrelationCalculator 1.01 (University of Michigan), a correlation matrix was calculated and then converted into Lasso–type partial correlations.

A .csv file containing Lasso partial correlations was entered into the Metscape application in the Cytoscape package to create an undirected graph. Positive interactions indicated co–occurrence or a mutualistic relationship between the biomass of the taxa, while negative interactions indicated predator–prey relationships or competition^22^. The graph only contains correlations that are statistically significant at p ≤ 0.05.

We used the edge–weighted spring–embedded layout with Pearson correlations as weights to plot the graphs showing the relationships between insect species in the three regions. In weighted graphs, the distance between nodes is defined as the sum of the weights^68^. Nodes in such a layout of a network are like physical objects that repel each other. Edges "repel" or "attract" nodes depending on the weight strength function (correlation). The position of nodes is set to minimise the sum of strengths (correlations) in the network^67^.

We have calculated values for the most important attributes of a network as a whole, which are used in studies on organism communities^22,23,69^. These were the number of neighbours, the nearest path, the clustering coefficient, network centralisation, network density and network heterogeneity. We used the following metrics to characterise the role of specific nodes (species) and connections between them: correlation coefficient (R), node degree centrality (NDC)^68^, node closeness centrality (NCC)^70^, node betweenness centrality (NBC) and edge betweenness centrality (EBC)^71^. NDC indicates the number of immediate neighbours of a species that are connected to that species. NCC is a metric for the speed with which information is passed from a given species to other taxa in the network. NBC represents the importance of a particular taxon, and EBC shows the importance of a particular relationship for the cohesion of the whole network. EBC represents the number of shortest paths crossing the edge of a graph. In our study, EBC identifies the importance of interactions between taxa for the cohesion of the network of the entire biocoenosis. The paper by Kruk and Paturej^22^ contains a more detailed description of the above metrics.

### Supplementary Information

Supplementary Table S1.

Supplementary Table S2.

Supplementary Information.

## Data Availability

All data generated or analysed as part of this study are included in this published article [and its supplementary information files].
