# Supplementary material for: Regional differences in water beetle communities networks settling in dystrophic lakes in northern Poland
Source: Sci Rep. 2023 Aug 5;13:12699. doi: 10.1038/s41598-023-39689-z (PMC10404283; doi:10.1038/s41598-023-39689-z)
Supplement: Supplementary file 1 — Supplementary Table S1. [file 41598_2023_39689_MOESM1_ESM.docx]

**Table S.1.** Quantitative occurrence of beetles in studied lake: E –ecological groups (L – lake and river species, T – tyrphophilous species, E – eurytopic species, A – psammophilous species); FFG – feeding functional groups, Stage of succession: O – oligohumic, M – mesohumic, P – polyhumic), Total – N – number of individuals,

|  |  |  | **Kashubian Lakeland** | | | | | | **Mazurian Lakeland** | | | | **Suwalki Lakeland** | **Total** |
| --- | --- | --- | --- | --- | --- | --- | --- | --- | --- | --- | --- | --- | --- | --- |
| **Species** | **E** | **FFG** | **O** | **O-M** | **M** | **M-P** | **P** | **Tot** | **M** | **M-P** | **P** | **Tot** | **P** |  |
| *Gyrinus aeratus Steph.* | L | Predators |  | 4 |  | 10 |  | 14 |  |  | 9 | 9 |  | 23 |
| *Gyrinus marinus Gyll.* | L | Predators |  | 1 |  |  |  | 1 |  |  |  |  |  | 1 |
| *Gyrinus paykuli* Ochs | E | Predators |  |  |  | 1 |  | 1 |  |  |  |  |  | 1 |
| *Gyrinus substriatus Steph.* | E | Predators |  |  |  |  |  |  | 2 |  | 1 | 3 | 1 | 4 |
| *Gyrinus suffriani* Scriba | E | Predators |  |  |  |  |  |  |  |  | 3 | 3 | 1 | 4 |
| *Haliplus confinis* Steph. | A | Polyphaga |  |  |  |  |  |  |  |  |  |  | 1 | 1 |
| *Haliplus fluviatilis Aubé* | L | Polyphaga |  |  |  |  |  |  |  |  | 3 | 3 |  | 3 |
| *Haliplus fulvicollis* Er. | T | Polyphaga |  |  |  |  |  |  |  |  | 3 | 3 |  | 3 |
| *Haliplus immaculatus* Gerh. | E | Polyphaga |  |  |  |  |  |  |  |  |  |  | 1 | 1 |
| *Haliplus ruficollis* (De Geer) | E | Polyphaga |  | 1 |  |  |  | 1 |  | 1 | 2 | 3 | 1 | 5 |
| *Noterus clavicornis* (De Geer) | E | Predators |  | 1 |  | 1 | 1 | 3 |  |  |  |  | 3 | 6 |
| *Noterus crassicornis* (O.F. Mȕll.) | E | Predators | 32 | 66 | 2 | 30 | 22 | 152 | 237 | 102 | 325 | 664 | 627 | 1443 |
| *Agabus affinis* (Payk.) | T | Predators |  | 1 |  |  |  | 1 |  |  |  |  | 3 | 4 |
| *Agabus congener* (Thunb.) | E | Predators |  |  |  |  |  |  | 1 |  |  | 1 |  | 1 |
| *Agabus labiatus* (Brahm) | T | Predators |  |  |  |  |  |  |  |  |  |  | 1 | 1 |
| *Agabus undulatus* (Schrank) | E | Predators |  | 6 |  |  |  | 6 |  |  |  |  |  | 6 |
| *Ilybius aenescens* Thoms. | T | Predators |  | 1 |  |  |  | 1 |  |  |  |  |  | 1 |
| *Ilybius ater (De Geer)* | T | Predators |  |  |  | 1 |  | 1 |  |  |  |  |  | 1 |
| *Ilybius fenestratus* (Fabr.) | L | Predators | 12 | 2 |  | 9 |  | 23 |  | 1 | 3 | 4 | 2 | 29 |
| *Ilybius fuliginosus* (Fabr.) | L | Predators |  | 1 |  |  |  | 1 |  |  | 1 | 1 |  | 2 |
| *Ilybius guttiger* (Gyll.) | T | Predators |  |  |  |  |  |  |  |  |  |  | 1 | 1 |
| *Ilybius obscurus* (Marsh.) | T | Predators |  |  |  |  |  |  |  | 2 |  | 2 |  | 2 |
| *Ilybius quadriguttatus* (Lacord.) | T | Predators |  |  |  |  |  |  |  |  | 2 | 2 |  | 2 |
| *Ilybius similis* Thoms. | T | Predators |  |  |  |  |  |  |  |  |  |  | 3 | 3 |
| *Ilybius subaeneus* Er. | T | Predators |  |  |  |  |  |  |  | 1 |  | 1 | 5 | 6 |
| *Colymbetes fuscus* (L.) | E | Predators |  |  |  |  | 2 | 2 |  |  |  |  |  | 2 |
| *Colymbetes paykuli* Er. | T | Predators |  |  |  |  |  |  |  |  | 2 | 2 |  | 2 |
| *Colymbetes striatus (L.)* | T | Predators |  |  |  |  |  |  |  |  | 9 | 9 |  | 9 |
| *Rhantus bistriatus* (Bergst.) | E | Predators |  | 1 |  |  |  | 1 |  |  |  |  |  | 1 |
| *Rhantus exsoletus (Forst.)* | E | Predators | 1 |  |  |  |  | 1 |  |  |  |  |  | 1 |
| *Rhantus grapi* (Gyll.) | E | Predators |  |  |  |  |  |  | 1 |  | 1 | 2 | 1 | 3 |
| *Rhantus latitans* Sharp | E | Predators |  | 1 |  |  |  | 1 |  |  |  |  | 7 | 8 |
| *Rhantus notaticollis* ( Aubé) | E | Predators |  |  |  |  |  |  |  |  |  |  | 1 | 1 |
| *Rhantus notatus* (Fabr.) | E | Predators |  |  |  |  |  |  |  | 1 | 2 | 3 | 1 | 4 |
| *Rhantus suturalis* (Mac L.) | E | Predators |  | 1 |  |  |  | 1 | 1 |  | 1 | 2 | 9 | 12 |
| *Acilius canaliculatus* (Nic.) | T | Predators |  |  |  |  | 4 | 4 | 10 | 5 | 15 | 30 | 21 | 55 |
| *Acilius sulcatus* (L.) | E | Predators | 1 |  |  |  | 1 | 2 | 14 | 2 | 23 | 39 | 8 | 49 |
| *Graphoderus bilineatus* (De Geer) | E | Predators |  |  |  |  |  |  |  |  |  |  | 3 | 3 |
| *Graphoderus cinereus* (L.) | E | Predators |  |  |  |  |  |  | 1 | 2 | 11 | 14 | 10 | 24 |
| *Graptodytes bilineatus* (Sturm) | E | Predators |  |  |  |  |  |  |  |  | 1 | 1 |  | 1 |
| *Cybister lateralimarginalis* (De Geer) | E | Predators |  |  |  |  |  |  |  |  | 11 | 11 |  | 11 |
| *Dytiscus dimidiatus* Bergst. | E | Predators |  | 1 |  |  |  | 1 |  |  |  |  |  | 1 |
| *Dytiscus lapponicus* Gyll. | T | Predators | 1 |  |  |  |  | 1 |  |  | 3 | 3 |  | 4 |
| *Dytiscus marginalis* (L.) | E | Predators |  |  |  |  | 1 | 1 |  | 1 | 1 | 2 | 3 | 6 |
| *Hydaticus aruspex* Clark | T | Predators |  |  |  |  |  |  |  | 1 |  | 1 |  | 1 |
| *Hydaticus modestus* Sharp | E | Predators |  |  |  |  |  |  |  |  |  |  | 1 | 1 |
| *Hydaticus seminiger* (De Geer) | E | Predators |  | 1 |  |  |  | 1 |  |  | 7 | 7 | 5 | 13 |
| *Nebrioporus canaliculatus* (Lacord.) | A | Predators |  |  |  |  |  |  |  | 1 |  | 1 |  | 1 |
| *Graptodytes pictus* (Fabr.) | E | Predators | 17 |  | 2 | 18 |  | 37 |  | 6 |  | 6 | 6 | 49 |
| *Hydroporus angustatus* (Sturm) | T | Predators | 1 | 4 |  |  | 1 | 6 | 5 | 2 | 5 | 12 | 20 | 38 |
| *Hydroporus dorsalis* (Fabr.) | E | Predators |  |  |  |  |  |  |  |  | 2 | 2 | 2 | 4 |
| *Hydroporus elongatulus Sturm* | T | Predators |  |  | 1 |  |  | 1 |  |  |  |  |  | 1 |
| *Hydroporus erythrocephalus* (L.) | T | Predators | 1 | 4 |  |  |  | 5 | 1 |  | 3 | 4 | 10 | 19 |
| *Hydroporus incognitus* Sharp | T | Predators |  | 1 | 13 |  |  | 14 | 6 |  |  | 6 | 4 | 24 |
| *Hydroporus melanocephalus* (Marsh.) | T | Predators |  |  | 1 |  |  | 1 |  |  |  |  |  | 1 |
| *Hydroporus memnonius* Nic. | T | Predators |  |  |  |  |  |  | 4 |  | 1 | 5 |  | 5 |
| *Hydroporus neglectus* Schaum | T | Predators |  |  |  |  | 1 | 1 | 8 | 8 | 26 | 42 | 19 | 62 |
| *Hydroporus notatus* Sturm | T | Predators |  |  |  |  |  |  | 1 |  |  | 1 | 1 | 2 |
| *Hydroporus obscurus* Sturm | T | Predators | 2 |  |  |  | 2 | 4 | 24 |  | 9 | 33 | 40 | 77 |
| *Hydroporus palustris* (L.) | E | Predators |  | 57 |  |  |  | 57 | 10 |  | 1 | 11 | 2 | 70 |
| *Hydroporus piceus* (L.) | T | Predators |  |  |  |  |  |  |  |  | 2 | 2 |  | 2 |
| *Hydroporus pubescens* (Gyll.) | T | Predators |  |  |  |  |  |  |  |  |  |  | 1 | 1 |
| *Hydroporus rufifrons* (Duft.) | E | Predators |  |  |  |  |  |  |  |  |  |  | 1 | 1 |
| Hydroporus scalesianus Steph. | T | Predators |  |  | 1 |  | 1 | 2 | 1 | 2 | 1 | 4 | 6 | 12 |
| Hydroporus tristis Payk. | T | Predators | 1 | 8 | 2 |  | 1 | 12 | 68 | 12 | 73 | 153 | 130 | 295 |
| *Hydroporus umbrosus* (Gyll.) | T | Predators |  |  |  |  |  |  | 12 |  |  | 12 | 10 | 22 |
| *Porhydrus lineatus* (Fabr.) | L | Predators |  |  |  |  |  |  | 1 | 1 | 5 | 7 | 7 | 14 |
| *Hygrotus decoratus* (Gyll.) | T | Predators |  |  |  |  |  |  |  |  | 12 | 12 | 14 | 26 |
| *Hygrotus inaequalis* (Schall.) | E | Predators | 1 | 2 |  |  |  | 3 | 2 | 2 | 14 | 18 | 19 | 40 |
| *Hygrotus impressopunctatus* (Schall.) | E | Predators |  |  |  |  |  |  | 2 | 2 | 1 | 5 | 10 | 15 |
| *Hyphydrus ovatus* (L.) | E | Predators | 6 | 6 | 1 | 1 |  | 14 | 3 |  | 10 | 13 | 63 | 90 |
| *Hydroglyphus pusillus* (Fabr.) | A | Predators |  |  |  |  | 1 | 1 | 2 | 1 |  | 3 | 70 | 74 |
| *Bidessus unistriatus* Goeze | E | Predators |  |  |  |  |  |  |  | 2 |  | 2 | 3 | 5 |
| *Laccophilus minutus* (L.) | E | Predators | 1 |  |  |  | 1 | 2 | 13 |  | 13 | 26 | 12 | 40 |
| *Helophorus fulgidicollis* Motsch. | A | Shredders |  |  |  |  |  |  |  |  |  |  | 2 | 2 |
| *Helophorus granularis* (L.) | A | Shredders |  |  |  |  | 1 | 1 |  |  | 1 | 1 | 3 | 5 |
| *Helophorus griseus* Herbst | A | Shredders |  |  |  |  | 1 | 1 |  |  |  |  |  | 1 |
| *Helophorus minutus* Fabr. | A | Shredders |  |  |  |  |  |  | 5 |  | 4 | 9 | 23 | 32 |
| *Helophorus pumilio* Er. | A | Shredders |  |  |  |  |  |  | 1 |  |  | 1 | 1 | 2 |
| *Hydrochus brevis* (Herbst) | E | Shredders |  |  |  |  |  |  |  |  |  |  | 2 | 2 |
| *Hydrochus crenatus* (Fabr.) | E | Shredders |  | 2 |  |  |  | 2 | 3 |  | 11 | 14 |  | 16 |
| *Hydrochus elongatus* (Schall.) | E | Shredders |  |  |  |  | 1 | 1 |  |  | 3 | 3 | 1 | 5 |
| *Hydrochus nitidicollis* Muls. | E | Shredders |  |  |  |  |  |  |  |  | 1 | 1 |  | 1 |
| *Anacaena limbata* (Fabr.) | E | Shredders |  | 11 |  |  |  | 11 |  |  | 4 | 4 |  | 15 |
| *Anacaena lutescens* (Steph.) | T | Shredders | 26 | 78 | 5 | 3 | 23 | 135 | 255 | 20 | 41 | 316 | 325 | 776 |
| *Berosus signaticollis* (Charp.) | E | Shredders |  |  |  |  |  |  |  |  |  |  | 1 | 1 |
| *Cymbiodyta marginella* (Fabr.) | T | Shredders |  |  |  |  |  |  |  |  | 4 | 4 |  | 4 |
| *Enochrus affinis* (Thunb.) | T | Shredders | 4 | 23 |  |  | 5 | 32 | 39 | 1 | 19 | 59 | 109 | 200 |
| *Enochrus coarctatus* (Gredl.) | T | Shredders |  | 5 |  | 2 | 24 | 31 | 22 | 7 | 54 | 83 | 73 | 187 |
| *Enochrus melanocephalus* (Ol.) | T | Shredders |  |  |  |  |  |  |  |  | 1 | 1 |  | 1 |
| *Enochrus ochropterus* (Marsh.) | E | Shredders |  | 6 |  |  | 7 | 13 | 33 | 3 | 29 | 65 | 34 | 112 |
| *Enochrus quadripunctatus* (Herbst) | E | Shredders |  |  |  |  |  |  | 1 |  |  | 1 | 19 | 20 |
| *Enochrus testaceus* (Fabr.) | E | Shredders | 1 | 2 |  |  |  | 3 |  |  |  |  | 3 | 6 |
| *Helochares obscurus* (O.F. Mȕll.) | A | Shredders | 33 | 23 |  | 1 | 4 | 61 | 45 | 1 | 20 | 66 | 65 | 192 |
| *Hydrobius fuscipes* (L.) | E | Shredders |  | 3 |  | 1 |  | 4 | 2 |  | 3 | 5 | 10 | 19 |
| *Hydrochara caraboides* (L.) | E | Shredders |  |  |  |  |  |  | 1 |  | 11 | 12 | 2 | 14 |
| *Laccobius minutus* (L.) | A | Shredders | 2 | 1 |  |  | 2 | 5 | 5 |  | 2 | 7 | 10 | 22 |
| *Coelostoma orbiculare* (Fabr.) | E | Shredders | 3 | 2 |  | 1 | 6 | 12 | 25 | 5 | 30 | 60 | 30 | 102 |
| *Cercyon convexiusculus* Steph. | E | Shredders |  | 1 |  |  |  | 1 |  |  |  |  |  | 1 |
| *Cercyon lateralis* (Marsh.) | E | Shredders |  |  |  |  |  |  |  |  |  |  | 1 | 1 |
| *Cercyon tristis* (Ill.) | E | Shredders |  |  |  |  |  |  |  |  | 2 | 2 |  | 2 |
| *Limnebius parvulus* (Herbst) | E | Grazer and scraper |  | 1 |  |  |  | 1 |  |  | 12 | 12 | 16 | 29 |
| *Ochthebius minimus* (Fabr.) | E | Grazer and scraper |  |  |  |  |  |  | 1 |  |  | 1 | 1 | 2 |
| *Hydraena palustris* Er. | T | Grazer and scraper |  |  |  |  |  |  |  |  | 5 | 5 |  | 5 |
| **Total** |  |  | **146** | **329** | **28** | **79** | **113** | **695** | **868** | **195** | **874** | **1937** | **1901** | **4533** |
