# Supplementary material for: Regional differences in water beetle communities networks settling in dystrophic lakes in northern Poland
Source: Sci Rep. 2023 Aug 5;13:12699. doi: 10.1038/s41598-023-39689-z (PMC10404283; doi:10.1038/s41598-023-39689-z)
Supplement: Supplementary file 2 — Supplementary Table S2. [file 41598_2023_39689_MOESM2_ESM.docx]

**Table S 2**. General characteristics of the lakes: % – contribution in the littoral zone (Sm – *Sphagnum* mat, Di – Diffuse macrophytes, De - Dense macrophytes, A – arenal zone), T – Stage of succession (O – oligohumic, M – mesohumic, P – polyhumic), S – number of species.

| Lake | Geographic N | Coordinates E | Area (ha) | Cover mat  (%) | % | | | | T | S |
| --- | --- | --- | --- | --- | --- | --- | --- | --- | --- | --- |
|  |  |  |  |  | Sm | Di | De | A |  |  |
| **Kaszuby Lakeland** | **Mazurian Lakeland** |  |  |  |  |  |  |  |  |  |
| Długie | 53°48’29’’ | 17°37’10’’ | 6.20 | 3 | 0.1 | 0.7 | – | 0.2 | O–M | 33 |
| Klimontek | 53°42’21’’ | 17°26’07’’ | 0.40 | 10 | 0.8 | 0.1 | – | 0.1 | M–P | 13 |
| Małe Gacno | 53°47’07’’ | 17°33’01’’ | 17.50 | 2 | 0.1 | 0.7 | – | 0.2 | O | 11 |
| Małe Łowne | 53°48’10’’ | 17°27’29’’ | 2.50 | 11 | 0.2 | 0.7 | – | 0.1 | M | 9 |
| Piecki | 53°54’44’’ | 17°33’17’’ | 6.00 | 11 | 0.3 | 0.3 | 0.1 | 0.3 | O–M | 4 |
| Sosnówek | 53°48’59’’ | 17°36’58’’ | 3.50 | 2 | 0.05 | 0.8 | 0.05 | 0.1 | O | 34 |
| Żabionek | 53°49’55’’ | 17°37’06’’ | 5.8 | 14 | 0.7 | 0.2 | 0.1 | – | P | 23 |
| **Mazurian Lakeland** |  |  |  |  |  |  |  |  |  |  |
| Bobrówko | 53°45’02’’ | 21°31’11’’ | 1.30 | 40 | 0.9 | 0.1 | –­­ | – | P | 3 |
| Borkowskie | 53°43’29’’ | 21°32’89’’ | 2.70 | 37 | 0.9 | 0.1 | – | – | P | 6 |
| Białe | 53°53’29’’ | 20°49’21’’ | 2.88 | 3 | 0.8 | 0.1 | 0.1 | – | M | 33 |
| Gryżewskie | 53°43’39’’ | 21°32’48’’ | 4.0 | 42 | 0.5 | 0.1 | 0.4 | 0.1 | M | 6 |
| Krucze Oko | 53°39’33’’ | 21°24’11’’ | 0.4 | 35 | 0.9 | 0.1 | – | – | P | 1 |
| Kruczy Staw | 53°39’30’’ | 21°24’14’’ | 2.08 | 15 | 0.9 | 0.1 | – | – | M–P | 4 |
| Jonkowo | 53°48’49’’ | 20°19’05’’ | 2.95 | 20 | 0.8 | 0.1 | 0.1 | – | M–P | 19 |
| Kociołek | 53°54’01’’ | 20°49’11’’ | 0.56 | 5 | 0.9 | 0.1 | – | – | M | 19 |
| Kruczek Duży | 53°39’33’’ | 21°24’11’’ | 4.24 | 20 | 0.9 | 0.1 | – | – | M–P | 3 |
| Kruczek Mały | 53°39’27’’ | 21°25’01’’ | 2.56 | 10 | 0.9 | 0.1 | – | – | M–P | 13 |
| Motylek | 53°40’16’’ | 20°05’46’’ | 3.30 | 39 | 0.8 | 0.2 | – | – | P | 27 |
| Żabie | 53°50’00’’ | 15°02’00’’ | 1.78 | 70 | 0.8 | 0.1 | 0.1 | – | P | 50 |
| **Suwalki Lakeland** |  |  |  |  |  |  |  |  |  |  |
| Suchar 1 | 54°05’07’’ | 23°00’54’’ | 1.12 | 31 | 0.8 | 0.2 | – | – | P | 30 |
| Suchar 2 | 54°05’13’’ | 23°01’03’’ | 2.60 | 32 | 0.8 | 0.1 | – | 0.1 | P | 20 |
| Suchar 3 | 54°05’18’’ | 23°01’18’’ | 0.45 | 39 | 0.8 | 0.2 | – | – | P | 34 |
| Suchar 4 | 54°05’22’’ | 23°01’19’’ | 1.04 | 44 | 0.8 | 0.2 | – | – | P | 37 |
| Suchar 5 | 54°05’20’’ | 23°01’57’’ | 2.14 | 75 | 0.9 | 0.1 | – | – | P | 46 |
| Suchar Wielki | 54°01’40’’ | 23°03’19’’ | 8.90 | 31 | 0.8 | 0.2 | – | – | P | 27 |
